# Population Genomics of the Blue Shark, *Prionace glauca*, Reveals Different Populations in the Mediterranean Sea and the Northeast Atlantic

**DOI:** 10.1111/eva.70005

**Published:** 2024-09-17

**Authors:** Agostino Leone, Sophie Arnaud‐Haond, Massimiliano Babbucci, Luca Bargelloni, Ilaria Coscia, Dimitrios Damalas, Chrystelle Delord, Rafaella Franch, Fulvio Garibaldi, David Macias, Stefano Mariani, Jann Martinsohn, Persefoni Megalofonou, Primo Micarelli, Natacha Nikolic, Paulo A. Prodöhl, Emilio Sperone, Marco Stagioni, Antonella Zanzi, Alessia Cariani, Fausto Tinti

**Affiliations:** ^1^ Department of Biological, Geological and Environmental Sciences (BiGeA), Laboratory of Genetics and Genomics of Marine Resources and Environment University of Bologna Ravenna Italy; ^2^ MARBEC – University of Montpellier, CNRS, Ifremer, IRD Sète France; ^3^ Department of Earth and Marine Sciences (DiSTeM) University of Palermo Palermo Italy; ^4^ NBFC, National Biodiversity Future Center Palermo Italy; ^5^ Department of Comparative Biomedicine and Food Science University of Padova Legnaro Italy; ^6^ Marine Institute Rinville, Oranmore Ireland; ^7^ European Commission, Joint Research Centre, Directorate D – Sustainable Resources Ispra Italy; ^8^ Hellenic Centre for Marine Research Institute of Marine Biological Resources & Inland Waters, Former US Base at Gournes Heraklion Crete Greece; ^9^ Department of Earth, Environmental and Life Sciences University of Genova Genova Italy; ^10^ Instituto Español de Oceanografía Centro Oceanográfico de Málaga Malaga Spain; ^11^ School of Biological and Environmental Sciences Liverpool John Moores University Liverpool UK; ^12^ Department of Zoology‐Marine Biology, Faculty of Biology National and Kapodistrian University of Athens Athens Greece; ^13^ Sharks Studies Center—Scientific Institute Massa Marittima Italy; ^14^ INRAE, Ecobiop, AQUA Saint‐pée‐sur‐Nivelle France; ^15^ Institute for Global Food Security, School of Biological Sciences Queen's University Belfast Belfast UK; ^16^ Department of Biology, Ecology and Earth Sciences University of Calabria Arcavacata di Rende Italy; ^17^ Laboratory of Marine Biology and Fisheries, Department of Biological, Geological and Environmental Sciences (BiGeA) University of Bologna Fano Italy

**Keywords:** blue shark, connectivity, genome scan, pelagic sharks, SNPs, stock differentiation

## Abstract

Populations of marine top predators have been sharply declining during the past decades, and one‐third of chondrichthyans are currently threatened with extinction. Sustainable management measures and conservation plans of large pelagic sharks require knowledge on population genetic differentiation and demographic connectivity. Here, we present the case of the Mediterranean blue shark (*Prionace glauca*, L. 1758), commonly found as bycatch in longline fisheries and classified by the IUCN as critically endangered. The management of this species suffers from a scarcity of data about population structure and connectivity within the Mediterranean Sea and between this basin and the adjacent Northeast Atlantic. Here, we assessed the genetic diversity and spatial structure of blue shark from different areas of the Mediterranean Sea and the Northeast Atlantic through genome scan analyses. Pairwise genetic differentiation estimates (*F*_ST_) on 203 specimens genotyped at 14,713 ddRAD‐derived SNPs revealed subtle, yet significant, genetic differences within the Mediterranean sampling locations, and between the Mediterranean Sea and the Northeast Atlantic Ocean. Genetic differentiation suggests some degree of demographic independence between the Western and Eastern Mediterranean blue shark populations. Furthermore, results show limited genetic connectivity between the Mediterranean and the Atlantic basins, supporting the hypothesis of two distinct populations of blue shark separated by the Strait of Gibraltar. Although reproductive interactions may be limited, the faint genetic signal of differentiation suggests a recent common history between these units. Therefore, Mediterranean blue sharks may function akin to a metapopulation relying upon local demographic processes and connectivity dynamics, whereby the limited contemporary gene flow replenishment from the Atlantic may interplay with currently poorly regulated commercial catches and large‐scale ecosystem changes. Altogether, these results emphasise the need for revising management delineations applied to these critically endangered sharks.

## 1 Introduction

Detecting population structure, at various spatial scales, in large pelagic marine fish species, such as sharks, is essential for the implementation of reliable species conservation and fisheries management plans. Ideally, stock boundaries must encompass groups of individuals with similar demographic or genetic connectivity that share similar responses to fishing and other external pressures. This is of particularly importance for the conservation of large apex predators which are K‐selected species with potentially no external ‘replenishment’ and, thus, vulnerable to mismanagement (Ying et al. 2011; Reiss et al. 2009; Ferretti et al. 2008). This is far from easy, as intermediate scenarios between panmixia and absence of gene flow (Waples and Gaggiotti 2006) are particularly difficult to discern in large pelagic species (Bailleul et al. 2018; Puncher et al. 2018; Rodríguez‐Ezpeleta et al. 2019). Mismatches between biologically independent entities and management units are also common, with negative effects on the conservation of population complexes managed as a single entity (Reiss et al. 2009). The identification of these boundaries is essential for an accurate estimation of individual stock delineation.

Given the semi‐closed sea nature of the Mediterranean, its level of connectivity with the Atlantic Ocean in several marine species has been investigated (e.g., Maroso et al. 2021; Rodríguez‐Ezpeleta et al. 2019; Puncher et al. 2018; Catarino et al. 2015; Patarnello, Volckaert, and Castilho 2007).

The Mediterranean Sea harbours a high percentage of threatened sharks and rays, with more than half of the species being threatened with extinction (Walls and Dulvy 2021). Overfishing, including bycatch (non‐target species caught incidentally), is the main cause of the decline of shark populations (Pacoureau et al. 2021; Dulvy et al. 2014, 2021), and as several sharks and rays are top predators, their demographic decline is expected to affect the functioning of marine ecosystems (Estes et al. 2011; Myers et al. 2007).

The blue shark *Prionace glauca*, L. 1758 is no exception. Besides being targeted by commercial fishing, this viviparous K‐selected species with an average generation time of 9.8 years in the North Atlantic (Cortés et al. 2015; Nakano and Stevens 2008) is a major bycatch of longline and driftnet fisheries (Parra et al. 2023; Megalofonou, Damalas, and Yannopoulos 2005).

As a result of the impact of fishing on blue shark global populations (Fowler et al. 2005), the species has been classified as globally ‘Near Threatened’ on the IUCN Red List (Rigby et al. 2019). More importantly, blue shark is classified as ‘Critically Endangered’ in the Mediterranean Sea (Sims et al. 2016), where high fishing pressure is associated with a dramatic decrease in estimated abundance over the last decades (Ferretti et al. 2008). Yet, the population genetic structure, the spatial dynamics and the level of connectivity of the Mediterranean blue shark with the Atlantic are still poorly understood, despite the importance of this information for the correct management of the species in the region.

Previously published tagging studies and the analysis of fisheries‐dependent data (Kohler, Casey, and Turner 1998; Kohler et al. 2002; Kohler and Turner 2008; Ferretti et al. 2008; Megalofonou et al. 2009) suggest that the vast majority of blue sharks tagged in the Mediterranean Sea were immature and remained in the tagging area, with no migration movements towards the adjacent southern areas of the Northeast Atlantic. The only exception was one subadult female that moved a short distance to reach the adjacent Northeast Atlantic area (Kohler et al. 2002).

These tag–recapture surveys, carried out from 1962 to 2000, suggest that North Atlantic blue sharks form a single stock, separate from the Mediterranean Sea stock, and that migratory movements within the Atlantic basin are quite frequent (Kohler et al. 2002).

The analysis of two mitochondrial markers highlighted an apparent lack of geographical differentiation between the Mediterranean and the Northeast Atlantic on the basis of haplotype networks (Leone et al. 2017). However, the use of Ф_ST_ integrating haplotype divergence detected significant genetic structure among four geographical groups, suggesting that the analysis of spatial genetic structure in relation to sex ratio and size could indicate some level of sex/age‐biased migratory behaviour (Leone et al. 2017).

On the contrary, distribution and behavioural data suggest widespread panmixia, and the first genetic data using microsatellites confirmed this hypothesis (Veríssimo et al. 2017; Vandeperre et al. 2014).

Genetic studies have been carried out on Atlantic and Pacific blue shark populations using microsatellites, suggesting restricted gene flow between oceans (Ussami et al. 2011; Fitzpatrick et al. 2010; Veríssimo et al. 2017). However, the analysis of juvenile specimens (<2 year) from Atlantic Ocean nurseries (Western Iberia, Azores and South Africa) using both mitochondrial and microsatellite markers reported a lack of genetic differentiation, suggesting the presence of a panmictic population in the whole Atlantic Ocean (Veríssimo et al. 2017).

Similar results were reported by Bailleul et al. (2018), with microsatellite data supporting the occurrence of a single panmictic worldwide blue shark population, except for hints of faint genetic differentiation of Mediterranean populations compared with Pacific populations. As the level of exchange required to maintain genetic homogeneity is much lower than that required to maintain demographic interdependency, particularly for large populations (Waples and Gaggiotti 2006), Bailleul et al. (2018) performed simulations suggesting that the apparent panmixia in blue shark could be explained by a genetic lag‐time effect. In other terms, demographic changes are not likely detectable using standard genetic analysis before a long transitional period of time (coined the ‘population grey zone effect’).

More recent worldwide scale population genomic studies detected a subtle but significant level of differentiation between the Mediterranean and the North Atlantic (*F*_ST_ comprised between 0.0007 and 0.0010; Nikolic et al. 2023). These results, including a handful of Mediterranean specimens, confirmed the hypothesis previously made by Bailleul et al. (2018) that a more granular genome‐representation approach would allow exiting the ‘grey zone of population differentiation’ and reveal genetic differentiation if present. Nevertheless, these recent studies only included a limited sample of Mediterranean origin, particularly in the Eastern part, which precludes a thorough understanding of microevolutionary dynamics in the basin. The International Commission for the Conservation of Atlantic Tunas (ICCAT), which assesses the blue shark stocks, manages the species as separate stocks in the Atlantic Ocean and Mediterranean Sea, solely based on the results of previous tagging studies with a limited number of sharks tagged in the Atlantic and recaptured in the Mediterranean Sea (ICCAT 2009; Fitzmaurice et al. 2005). However, the need for more data to better delineate stock boundaries has been stressed (ICCAT 2023).

As the blue shark population structure within the Mediterranean Sea remains largely unknown, this work aimed to fill this knowledge gap, while also shedding further light on the connectivity between the Atlantic and Mediterranean—using a large set of genome‐wide SNPs—with the aim to contribute to the improved management and conservation of this species, and further expanding our understanding of how marine populations are formed and maintained.

## 2 Materials and Methods

### 2.1 Sampling

**FIGURE 1:**
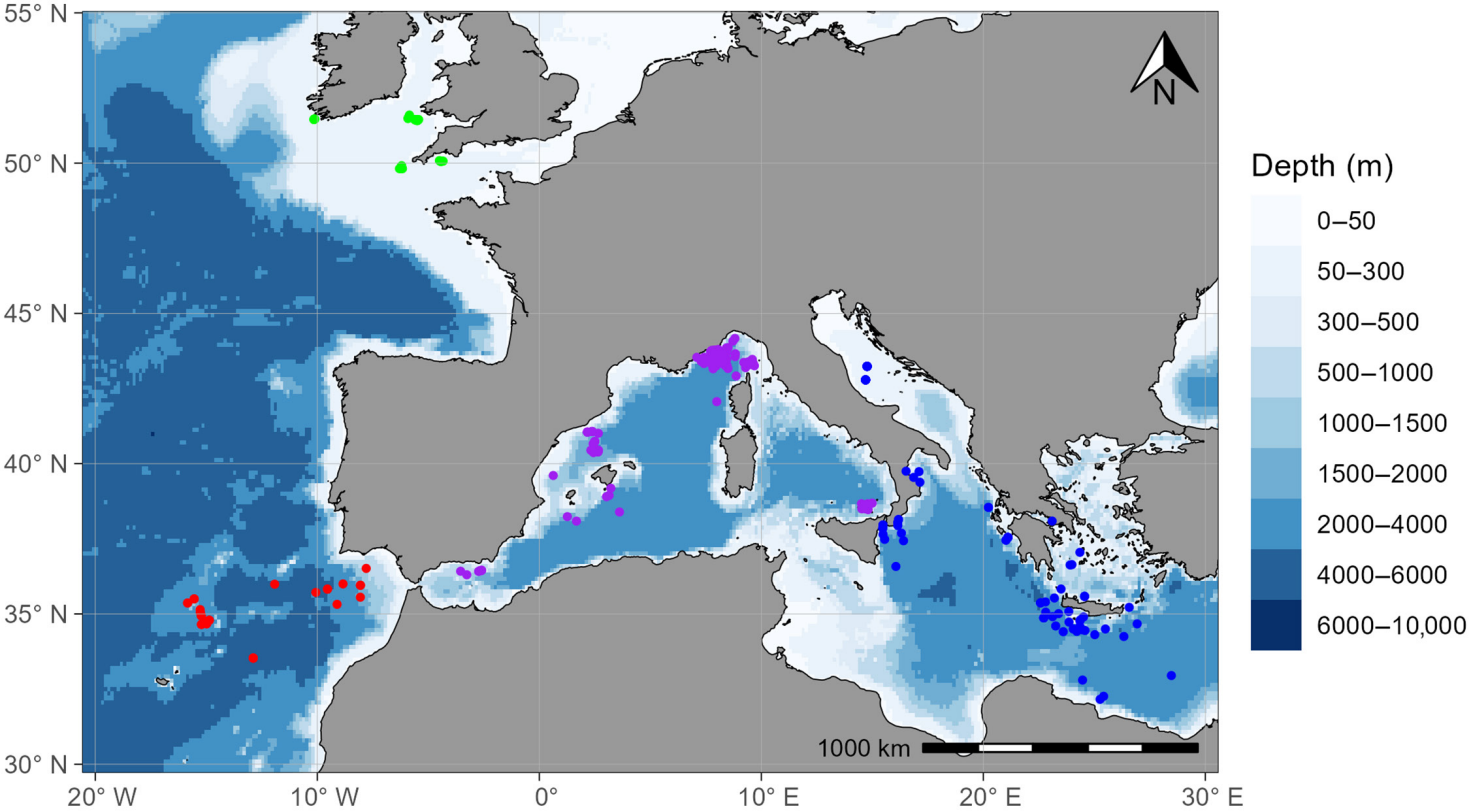
Sampling locations of blue sharks in the Celtic Sea (green dots), North Eastern Atlantic (red dots), Western Mediterranean (purple dots) and Eastern Mediterranean (blue dots). Blue shading indicates bathymetry (i.e., depth, in metres).

A total of 291 individuals were sampled in four areas, mostly as bycatch from commercial fisheries (Figure 1; Appendix S4): the Mediterranean (East Mediterranean, EMED: *n* = 111; West Mediterranean, WMED: *n* = 116), adjacent Northeast Atlantic areas from Gibraltar to Azores (Northeast Atlantic, EATL: *n* = 34) and from Southern Ireland and Great Britain (Celtic Sea, CELT: *n* = 30).

Muscle or skin tissue samples (ca 0.1–0.2 g) were collected using sterile scissors or tweezers and stored in 96% ethanol at −20°C. Specimens biological data as fork Length (in cm) and sex (female/male) as well as sampling data such as fishing date, geographical coordinates (longitude/latitude) and depth (in m) were collected whenever possible (Appendix S4).

### 2.2 Genomic Libraries Preparation and Sequencing

Genomic DNA (gDNA) was extracted using a modified salting‐out extraction protocol (Cruz et al. 2017). A modified ddRAD sequencing protocol was used to simultaneously genotype individuals at thousands of SNPs (Peterson et al. 2012; Brown et al. 2016).

Three ddRAD libraries were constructed, including individuals from different geographical areas distributed across three different libraries (Table S2) to avoid library bias. Briefly, for each individual, a standard quantity of 30 ng of gDNA was digested with *Sbf*I and *Sph*I (0.43 U of each, New England Biolabs). P1 and P2 barcoded adapters, compatible with the *Sbf*I and *Sph*I overhangs respectively, were mixed with T4 *ligase* and added to each sample. After enzyme heat inactivation, individual samples were pooled and cleaned up with MinElute PCR Clean Up Kit (Qiagen, Venlo, Netherlands).

Each library was run on an agarose gel (1.1%), to select fragments of 200–300 bp. Size‐selected DNA was then extracted from the gel. The eluted library was PCR amplified with generic P1 and P2 complementary primers after optimising the PCR conditions. The amplified library was purified using AMPure XP Magnetic Beads (Beckman Coulter, Pasadena, California, USA). Two reference individuals were included as replicates in each library to assess the sequencing/genotyping error rate. The obtained ddRAD libraries were paired‐end (PE) sequenced in three lanes using an Illumina HiSeq 4000. Demultiplexed reads are available on the NCBI Short Read Archive BioProject PRJNA1053301.

### 2.3 Bioinformatic Analysis and Loci Filtering

Raw sequencing data were checked for quality using FASTQC (version 0.11.8, Andrews 2010). Reads were demultiplexed using the program ‘process_radtags’ implemented by STACKS v. 1.42 (Catchen et al. 2011, 2013) avoiding ‐c and ‐q parameters, as suggested by the dDocent pipeline manual. The dDocent pipeline (www.ddocent.com; Puritz et al., 2014a, 2014b) was then used for reference construction, mapping reads and SNP calling. The pipeline dDocent has been specifically designed to analyse ddRADseq data of marine species, which are often characterised by high diversity and low differentiation (Puritz, Gold, and Portnoy 2016; Hollenbeck et al. 2017).

Characterising genotype data without the help of a reference genome presents several challenges, such as the pipeline trade‐off between splitting or lumping alleles into different clusters or a single locus, inflating homozygosity and heterozygosity, respectively. Similar issues have been addressed at the clustering step level using a high sequence similarity, from which a consensus sequence is derived. Additionally, haplotyping informative variants identified by dDocent using the rad_haplotyper.pl script by Willis et al. (2017) allowed for resolving any artificial clustering due to physical linkage between SNPs within locus typical at low levels of divergence among populations (Figures S1–S4). The haplotyping post‐clustering step mitigated also the effect of high levels of repeats and duplications expected in shark genomes.

Detailed assembly, SNP calling and filtering steps are described in the Appendices S1 and S2. Genomic data were then converted to the appropriate file format for subsequent population genetic analysis with PDGSpider (Lischer and Excoffier 2012). The final SNPs dataset was screened for outlier loci with three different approaches: the software Bayescan v. 2.1 (Foll and Gaggiotti 2008), the packages *pcadapt* (Luu, Bazin, and Blum 2017) and OutFLANK (Whitlock and Lotterhos 2015), implemented in the R environment version 4.0.5 (R Core Team 2021). See Appendix S3 for a detailed explanation of each of the three genome scan methods for outlier detection. All the resulting outlier loci were annotated for specific functions by matching the SNP flanking regions against the GenBank database (www.ncbi.nlm.nih.gov/genbank/) using BLAST (Altschul et al. 1990), and then removed from the dataset, producing a neutral loci dataset used for downstream analysis.

### 2.4 Population Genetics Analysis

Basic statistics of genetic diversity, heterozygosity, homozygosity and Hardy–Weinberg test were computed using the *diveRsity* R package (Keenan et al. 2013).

Genetic differentiation and population structure were inferred using three distinct families of approaches. First, pairwise *F*_ST_ and relative *p*‐values, following the Weir and Cockerham model (1984), were computed using the *StAMPP* R package (Pembleton, Cogan, and Forster 2013). Second, principal components analysis (PCA) and discriminant analysis of principal components (DAPC) were performed using the R package adegenet (Jombart 2008; Jombart, Devillard, and Balloux 2010; Jombart and Ahmed 2011) and plotted using the *ggplot2* package (Wickham 2016). Third, the genetic ancestry of each individual was estimated using the admixture model as implemented in the Bayesian clustering approach in STRUCTURE version 2.3.4 (Pritchard, Stephens, and Donnelly 2000). Results were obtained for *K* values (i.e., number of distinct genetic clusters) set from 1 to 5, and from 300,000 iterations following a burn‐in period of 100,000 iterations. The output from each *K* value (*K* from 1 to 5) was examined with (Jakobsson and Rosenberg 2007) to identify common modes, and results were plotted using DISTRUCT (Rosenberg 2004). The value of *K* that best fits the data was identified according to the Evanno method (Evanno, Regnaut, and Goudet 2005), as implemented in StructureHarvester (Earl and vonHoldt 2012), and according to Puechmaille (2016).

A Mantel test was used to test for isolation by distance per population. Four geographical points were chosen to be representative of the Celtic Sea, Northeast Atlantic, Western Mediterranean and Eastern Mediterranean (see Appendix S1 for details), and the minimum distance possible by seaway (Figure S5) was estimated using the R package *marmap* (Pante and Simon‐Bouhet 2013).

## 3 Results

Among the 291 specimens initially collected (Appendix S4), the sex of 263 individuals (118 males and 145 females) was determined, while for 28 individuals, no information was gathered, and after selecting for DNA extractions that met the quality standard for RAD sequencing, libraries were built and sequenced for a total of 212 blue sharks, plus four replicates (*n* = 216). This led us to discard 79 samples with poor preservation state that did not permit to obtain the high‐quality DNA extraction required by the protocol. Steps with sample selection and discard due to quality post‐sequencing are detailed in the Appendix S1.

After the demultiplexing, trimming and sample selection of the 3 ddRAD libraries (Pg_ddRAD01, Pg_ddRAD02 and Pg_ddRAD03), the number of retained reads per individual ranged from 688,054 to 66,488,950, with an average of 8,685,731 reads and a total of 1763 million of reads (detailed steps in Appendix S1). The dDocent pipeline identified 56,004 SNPs. After processing and filtering, the resulting dataset consisted of 14,729 SNPs and 203 blue shark individuals, distributed in the four geographical areas (CELT, EATL, WMED and EMED; Table S2) and across different years (2003–2016; Table S3).

Of these 14,729 SNPs, no SNPs were identified as outliers by BayeScan, 1 by OutFLANK and 15 SNPs by *pcadapt*, representing in total 0.11% of the retained SNPs. After removing these outliers, a final dataset in vcf format of 14,713 SNPs was created. Annotation of each of the outlier SNPs is available in Table S4.

Overall, the allelic richness observed was higher in the Mediterranean Sea than the Atlantic Ocean (Table 1). Heterozygosity values were similar among localities (Table 1). Significant Hardy–Weinberg disequilibrium and heterozygote deficiency were observed in the Western Mediterranean sample. The highest value of heterozygosity was observed in the Eastern Mediterranean samples (0.159), whereas the lowest value was observed in the two Atlantic samples (0.151) (Table 1).

**TABLE 1:** Genetic diversity estimates of blue sharks per geographic areas—Allelic richness (Ar) with the low and high CI, number of individuals (Nb), observed heterozygosity (Hobs), expected heterozygosity (Hexp), unbiased expected heterozygosity (Hexp_un), inbreeding coefficient (*F*_IS_) with the low and high CI on *F*_IS_ wrapper, *p*‐values from chi‐squared test for goodness‐of‐fit to Hardy–Weinberg equilibrium globally (hwe_glb), test significance for directional HWE on homozygote and heterozygote deficiency (hwe_hom; hwe_het).

	Ar	Nb	Hobs	Hexp	uexp_het	*F*_IS_	hwe_glb	hwe_hom	hwe_het
CELT	1766 (1727–1801)	28	0.151	0.163	0.166	0.054 (0.028–0.046)	1.000	1.000	1.000
EATL	1782 (1745–1816)	33	0.151	0.163	0.166	0.059 (0.037–0.051)	1.000	1.000	1.000
WMED	1834 (1806–1857)	88	0.152	0.166	0.167	0.077 (0.064–0.071)	0.000	1.000	0.000
EMED	1818 (1785–1846)	54	0.159	0.167	0.169	0.048 (0.021–0.050)	1.000	1.000	1.000

Pairwise *F*_ST_ values were low but significant for most comparisons after false discovery rate correction for multiple tests (Benjamini and Yekutieli 2001; Benjamini and Hochberg 1995; Table 2), with the exception of the comparisons between the Celtic Sea and either the Northeast Atlantic or the Western Mediterranean.

**TABLE 2:** Pairwise *F*_ST_ values (below diagonal) and associated *p*‐values (above diagonal) between blue shark samples based on the 14,713 neutral SNPs.

	CELT	EATL	WMED	EMED
CELT	\	0.1286	0.0883	0
EATL	0.00035	\	0.0097	0
WMED	0.00030	0.00043*	\	0
EMED	0.00170*	0.00152*	0.00068*	\

Abbreviations: CELT, Celtic Sea; EATL, Northeast Atlantic; EMED, Eastern Mediterranean; WMED, Western Mediterranean.

* Values significant after false discovery correction for multiple tests.

**FIGURE 2:**
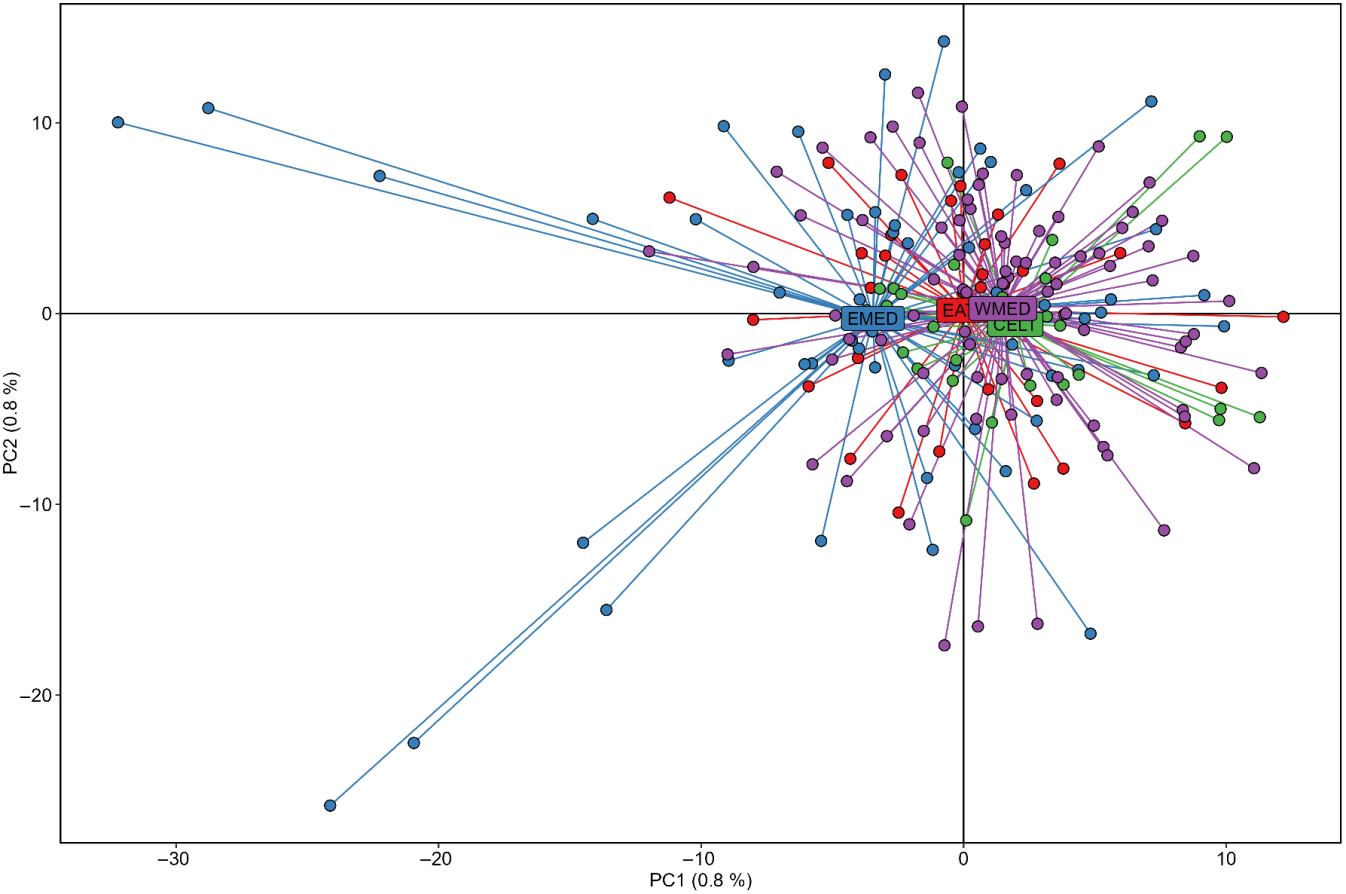
Analysis of the principal component analysis (PCA) plot using 14,713 neutral SNPs dataset. CELT, Celtic Sea (green); EATL, Northeast Atlantic (red); EMED, Eastern Mediterranean (blue); MED, Western Mediterranean (purple).

**FIGURE 3:**
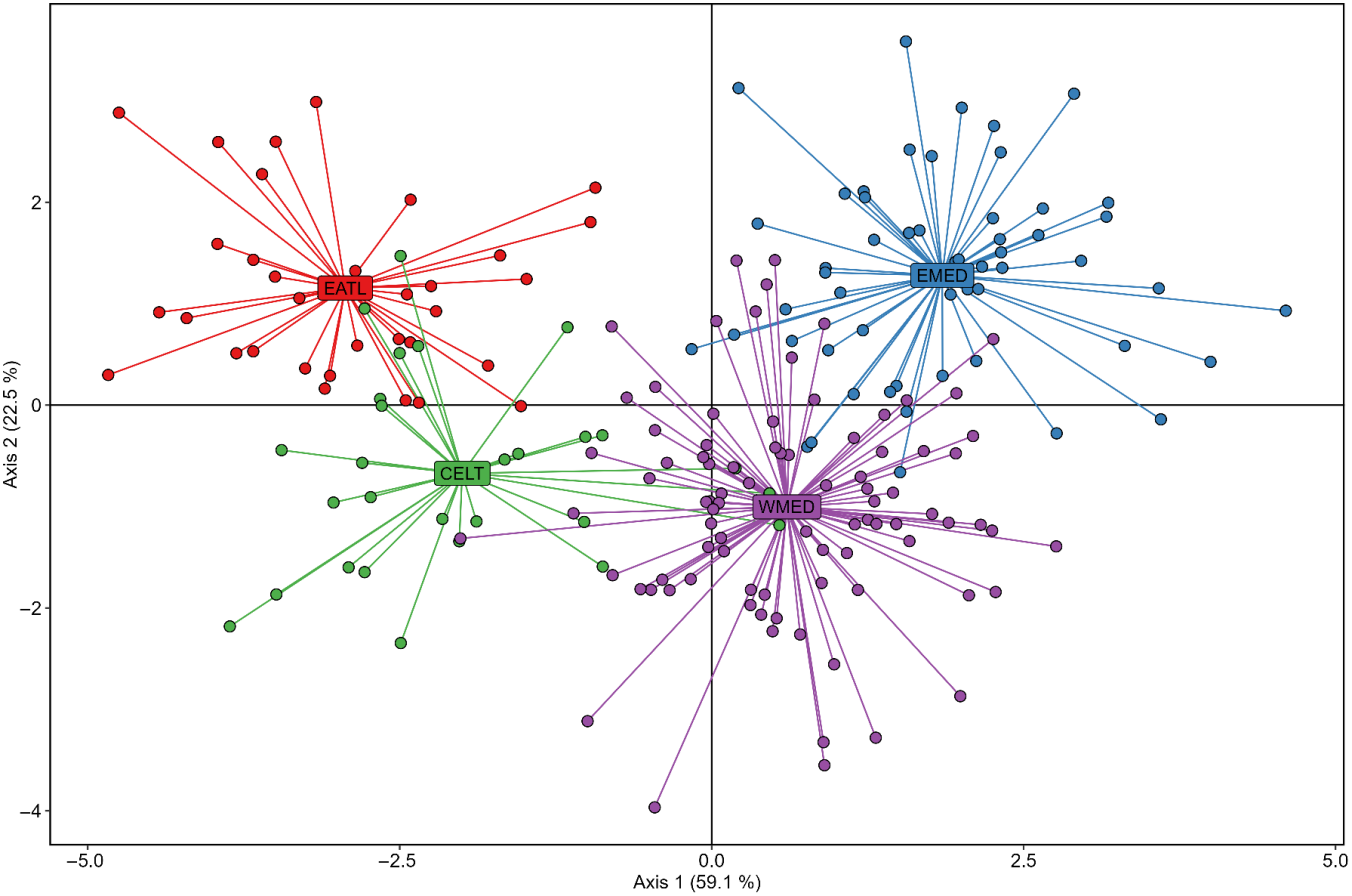
Discriminant analysis of principal component (DAPC) using 14,713 neutral SNPs dataset with a priori number of clusters of four. CELT, Celtic Sea (green); EATL, Northeast Atlantic (red); EMED, Eastern Mediterranean (blue); WMED, Western Mediterranean (purple).

**FIGURE 4:**
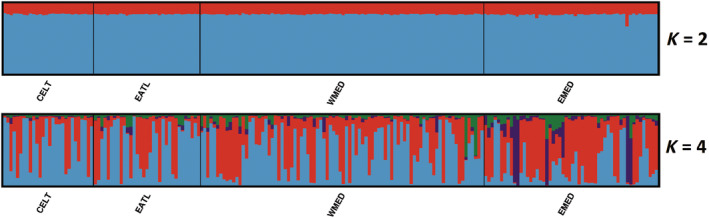
Resulting plot of the genetic clustering using STRUCTURE software for *K* = 2 and 4 as suggested by Puechmaill and Evanno's method respectively. CELT, Celtic Sea; EATL, Northeast Atlantic; EMED, Eastern Mediterranean; WMED, Western Mediterranean. Each bar on both plots represents the same individual.

Overall, multivariate PCA and DAPC analyses did not show any clear pattern of genetic structure among areas, despite a few Eastern Mediterranean individuals being genetically different from the rest (Figures 2 and 3). Similarly, the STRUCTURE Bayesian clustering, using the best *K* values according to the Puechmaille and Evanno methods (*K* = 2 and 4, respectively), showed no clear geographic clustering when *K* = 2, yet highlighted a few well‐differentiated individuals from the Eastern Mediterranean (Figure 4), in agreement with the PCA (Figure 2), while the DAPC more closely reflected the results observed with the pairwise *F*_ST_ analysis (Figure 3, Table 2).

A significant correlation between geographical and genetic distance, expressed as pairwise *F*_ST_, was detected through the Mantel test performed on the four geographical regions (Mantel statistic *r* = 0.7790, *y* = −0.00031 + 4e‐07*x*, *R* ^2^ = 0.61, *p* = 0.0417, Figure S6).

## 4 Discussion

Our study reveals the existence of subtle yet significant genetic differentiation between the Mediterranean and the Northeast Atlantic blue shark populations, confirming the Mediterranean singularity recently reported by Nikolic et al. (2023).

Our findings also suggest some substructure within the Mediterranean. These results contrast with previous studies based on low‐density genotyping, where no departure from large‐scale panmixia was detected in the entire Northeast Atlantic and Mediterranean areas (Bailleul et al. 2018; Veríssimo et al. 2017).

Furthermore, the use of larger sample sizes, including both adult and juvenile specimens, and denser sampling in the Mediterranean allowed the present study to highlight a faint but significant genetic differentiation between Western and Eastern Mediterranean groups (Table 2, Figures 2, 3, 4).

These results support the phylogeographic signal previously suggested based on mitochondrial DNA (Leone et al. 2017).

The limited heterozygote deficiency and *F*_IS_ values in our study are comparable to results obtained by Bailleul et al. (2018) using microsatellites. When comparing our findings to those obtained with SNPs by Nikolic et al. (2023), we observed lower values of *F*_IS_ in both Northeast Atlantic and Mediterranean areas (Table 1).

The genetic diversity of subsampled groups (Figure S7) confirms the patterns observed in Table 1 (Table S5), and pairwise *F*_ST_ values among subgroups confirm the significant differentiation of the Eastern Mediterranean blue sharks. Some comparisons, however, show nonsignificant values after correction for multiple test, possibly due to the limited sample size and associated statistical power of split groups.

Interestingly, the pairwise value between the Eastern Ionian Sea verses Adriatic Sea within the Eastern Mediterranean is still significant. However, these results may also be affected by low sample size, and more samples are needed to better resolve any other substructuring within the Mediterranean Sea (Table S6).

Similar to Nikolic et al. (2023), these significant *F*_ST_ values were accompanied by a lack of clear clustering pattern (when using multivariate and model‐based clustering methods), likely due to the low genetic signal of differentiation.

A significant exception in the present study is the remote position of some eastern Mediterranean individuals that seem to be well differentiated from all others (Figures 2, 3, 4 **)**, associated with higher *F*_ST_ values between the Eastern Mediterranean and all other areas (Table 2). Of these divergent individuals, three are from the Adriatic Sea, one from the Eastern Ionian Sea and one from Crete (Figure S7). The amount of divergence observed in these Eastern Mediterranean specimens may also suggest cases of Lessepsian migration from the Red Sea by this species, although this has never been reported. Such migrations are more commonly associated with small bony fishes and invertebrates (via ships/cargos), but have also been observed in elasmobranchs, such as *Carcharhinus melanopterus* and the *Carcharhinus brevipinna* (Bradai, Saidi, and Enajjar 2012). A dedicated study with larger sample sizes, including samples from the Red Sea, would be necessary to test this hypothesis.

Even removing the five most divergent specimens from the Eastern Mediterranean (see Appendix S1 for details), the overall genetic diversity and divergence do not change significantly (Tables S6 and S7). This suggests that the genetic structure observed in the present study is not the result of just a few divergent individuals alone, but rather the result of a genuine, subtle population structuring of blue shark populations within the Mediterranean Sea.

The genetic divergence of the Eastern Mediterranean sharks is also observed in split groups within the Mediterranean Sea (Figure S7) in both PCA and in DAPC analysis using a priori number of groups, supporting genetic differentiation within the Mediterranean (Figures S8 and S9). The correct number of clusters cannot be ascertained through the successive K‐means as in adegenet (Jombart 2008; Jombart, Devillard, and Balloux 2010; Jombart and Ahmed 2011), probably due to the subtle signal detected. In fact, generalised linear models applied on the relationship between clustering success and *F*_ST_ values on simulated data, examining the influence of a priori versus *de novo* group designations in DAPC analysis, highlight that the successive K‐means method does not reliably detect signal when *F*_ST_ between groups is not very high, particularly for large pelagic species (<0.1). This pleads for the use of a priori number of clusters based on the knowledge of the biology and behaviour of species under study (Miller, Cullingham, and Peery 2020).

Recent observations relating ecological data on blue shark distribution showed that large females may be more tolerant to cooler waters (Druon et al. 2022). This raises questions about the influence of sex on the spatial distribution of genetic diversity, as previously suggested based on mitochondrial phylogeography (Leone et al. 2017).

In the present study, the Celtic Sea is the only area where such a sex ratio (and life stage) bias is observed, as the majority of Celtic specimens sampled are large females. However, a larger dataset and a wider range of sampling will be needed to better investigate the relationship between genetic structure and sex.

Long‐term (four decades) tagging studies suggest that the large majority of blue sharks tagged in the Mediterranean Sea are immature and remain in the tagging area, avoiding movements towards the adjacent Northeast Atlantic. The only exception is one subadult female that moved a short distance to reach the adjacent Northeast Atlantic area (Kohler et al. 2002). Similarly, on the other side of the Strait of Gibraltar, only one adult male tagged in the Northeast Atlantic has been recaptured in the Mediterranean Sea (Kohler et al. 2002).

Telemetry data from blue sharks equipped with satellite tagging in the Western Mediterranean suggest a lack of connectivity with the Northeast Atlantic and with the adjacent Eastern Mediterranean blue sharks (Poisson et al. 2024). Altogether, these observations indicate a limited level of exchange among those areas, reflecting weak differentiation between these major basins (Northeast Atlantic, Western and Eastern Mediterranean; Nikolic et al. 2023; present study).

Furthermore, the result from the Mantel test is consistent with the existence of an isolation by distance in blue sharks, which implies non‐random mating and restricted gene flow among individuals from different sampled locations (see Results and Figures S5 and S6).

The lack of panmixia within the Mediterranean Sea may be explained by the environmental factors of the western and eastern Mediterranean, respectively. In fact, the Mediterranean Sea is characterised by different seas with very different oceanographic conditions (Tanhua et al. 2013). An environmental niche and habitat analysis of the blue shark on a global scale highlighted how biotic and abiotic factors may shape blue shark population distribution (Druon et al. 2022). In other pelagic species with similar spatial ecology, such as swordfish (*Xiphias gladius*), significant genetic structure has been observed between the Mediterranean Sea and the Atlantic Ocean, and within the Mediterranean Sea (Righi et al. 2020; Viñas et al. 2010).

Philopatric behaviour was suggested to be the main driver of swordfish population differentiation within the Mediterranean Sea because of distinct phylogeographic histories of populations in the eastern and the western Mediterranean basins, maintained by contemporary life‐history traits (Viñas et al. 2010).

Evidence of philopatry and regional site fidelity has been observed in blue sharks, with interannual resighting of blue sharks in the same spots in the mid‐North Atlantic (Fontes et al. 2024; Vandeperre et al. 2014). This philopatric behaviour, in combination with local demographic dynamics and potential site fidelity, may have shaped the current population differentiation of the blue shark between the Northeast Atlantic and the Mediterranean, and within the Mediterranean Sea.

### 4.1 Evolutionary Perspective of Subtle Genetic Structure

Accounting for the limited dispersal through the Gibraltar Strait observed with tagging data, the allele frequencies among even distant locations can be maintained at similar levels by very few migrants per generation. This can partially mask the existence of different demographic stocks (Palsbøll, Bérubé, and Allendorf 2007). In fact, even low migration rates, combined with a relatively large effective population size, can mask the existence of two demographically independent populations, suggesting a near‐panmictic scenario (Waples and Gaggiotti 2006).

This observed pattern could be explained by a marine metapopulation model as proposed by Kritzer and Sale (2004), in which genetic drift and gene flow determine ‘the dynamics of local populations strongly dependent upon local demographic processes, but also influenced by a nontrivial element of external replenishment’. If the dynamics of each potential population can be modelled per se (i.e., neglecting any potential external influence), the metapopulation scenario is not appropriate (Kritzer and Sale 2004). Otherwise, if the potential populations dictate their own population dynamics together with an external replenishment that cannot be ignored, then the metapopulation scenario is appropriate (Kritzer and Sale 2004). In these cases, it is the amount of demographic connectivity among potential populations set by migrant individuals that determine whether they form a metapopulation or not, and the rate of gene flow among units will determine the shape and fate of a given metapopulation complex and its components.

However, in the presence of small values of genetic differentiation, such as the *F*_ST_ values observed in the present study, the amount of gene flow is difficult to estimate under an island model of migration. This difficulty arises because of the relationship between *F*_ST_ and number of migrants among populations per generation (Lowe and Allendorf 2010). Furthermore, many biological assumptions necessary to estimate the gene flow under an island model of migration, are unrealistic and will be violated (Whitlock and McCauley 1999).

A metapopulation model has been used to explain the recent decline observed in three species of sharks when assuming unstructured demographic models, with the presence of a neglected population structure (Lesturgie, Planes, and Mona 2021). Beyond speculating about the existence of a metapopulation structure, even faint but significant genetic structure implies limited demographic exchange between populations. This is evident in the results observed in the present study and is in line with the stronger signal recently reported between the Atlantic and the Mediterranean by Nikolic et al. (2023). Furthermore, the pattern of isolation by distance (Figure S6) and those two concordant studies increase the confidence in the biological relevance of such subtle, yet significant, genetic structure (Palumbi 2003).

Based on the above results, the Mediterranean and Northeast Atlantic populations should be considered demographically independent, subject to area‐related population processes and different vulnerabilities to exploitation. Furthermore, even within the Mediterranean Sea (western Mediterranean vs. eastern Mediterranean), there is evidence of substructuring, with the presence of at least two subpopulations with independent demographic dynamics.

### 4.2 Management Implications of Multiple Discrete Population

The small number of sharks tagged in the Atlantic and recaptured in the Mediterranean Sea led management organisations to consider the Mediterranean as a separate stock (Kohler and Turner 2008; ICCAT 2005; Fitzmaurice et al. 2005; Kohler et al. 2002). For stock assessment purposes, separate analyses have been carried out for the North Atlantic and the Mediterranean for more than a decade. The ICCAT Sub Committee on bycatches assumed three different stocks in the North Atlantic, South Atlantic and Mediterranean (ICCAT 2005). The limited amount of tagging data made the separation of the Northeast Atlantic and Mediterranean blue shark in two different stocks a precautionary approach, as limited data from the Mediterranean blue shark were available. There was thus an acknowledged need for targeted studies to fill the knowledge gap about the existence of two separated populations on both sides of the Strait of Gibraltar (ICCAT 2016), a gap now filled through population genomics (present study; Nikolic et al. 2023), confirming the validity of this precautionary approach.

The present study may also serve to update future stock assessment and management plans. In fact, the genetic differentiation with significant *F*_ST_ values supports the existence of independent demographic entities for the blue shark within the Mediterranean as well, calling for a revision of recognised management units. The present study, echoing results from Nikolic et al. (2023), confirms the importance of using genome‐wide markers and dedicated sampling design in resolving the population genetic structure of the Northeast Atlantic and Mediterranean blue shark populations, especially considering the potential ‘grey zone’ effect in studies based on a handful of molecular markers (Bailleul et al. 2018).

Another possible area of future research would be to increase the sample size in the Mediterranean and in the Atlantic Ocean, including also the westernmost and the easternmost distribution of blue sharks from the Atlantic and Mediterranean Sea. This would clarify the relationships between western Atlantic and eastern Atlantic blue sharks with those from the western and eastern Mediterranean Sea. This is especially important in light of the extensive transatlantic migration observed, with consequent gene flow that follows.

A similar pattern of genetic differentiation within the Mediterranean has been reported thus far for the benthic small‐spotted catshark using both mitochondrial and microsatellite markers (Gubili et al. 2014; Kousteni et al. 2014; Melis et al. 2023), and in the black mouth catshark using microsatellite markers (Di et al. 2022). The significant differentiation observed in blue sharks between the Eastern and the Western Mediterranean suggests that the presence of discrete populations within the Mediterranean may also extend to pelagic sharks. Given the implications of such independence for the management of exploited or impacted populations, extending this study to other chondrichthyan species would be important for the conservation of these often declining groups.

## Conflicts of Interest

The authors declare no conflicts of interest.

## Supporting information


Appendix S1.

Appendix S2.

Appendix S3.

Appendix S4.

## Data Availability

All the sampling data are available on https://data.europa.eu/89h/57265ee1‐4c57‐49b1‐b456‐10758f161594 and in the Appendix S4. Demultiplexed reads are deposited on the NCBI Sequence Read Archive (SRA) under BioProject PRJNA1053301. Data filtering commented steps, codes and scripts are available in the Appendices S2 and S3 and in the GitHub repository https://github.com/leoneago/MedBlueSGen.
