# Supplementary material for: Population Genomics of the Blue Shark, Prionace glauca, Reveals Different Populations in the Mediterranean Sea and the Northeast Atlantic
Source: Evol Appl. 2024 Sep 17;17(9):e70005. doi: 10.1111/eva.70005 (PMC11408569; doi:10.1111/eva.70005)
Supplement: Supplementary file 1 — Appendix S1. [file EVA-17-e70005-s004.docx]

**Supporting Information for:**

**Population genomics of the blue shark, *Prionace glauca*, reveals different populations in the Mediterranean Sea and the North East Atlantic**

*Agostino Leone*, Sophie Arnaud-Haond, Massimiliano Babbucci, Luca Bargelloni, Ilaria Coscia, Dimitrios Damalas, Chrystelle Delord, Rafaella Franch, Fulvio Garibaldi, David Macias, Stefano Mariani, Jann Martinsohn, Persefoni Megalofonou, Primo Micarelli, Natacha Nikolic, Paulo A. Prodöhl, Emilio Sperone, Marco Stagioni, Antonella Zanzi, Alessia Cariani, Fausto Tinti**

**Supplementary Text: Reads processing and variants filtering**

**Methods**

Fastq files were demultiplexed and filtered with the program ‘process_radtags v. 1.42’ from the Stacks package (<http://creskolab.uoregon.edu/stacks/>; Catchen et al. 2011, 2013). For each library, data were separated according to inline barcodes and specifying the restriction site (*Sbf*I and *Sph*I, respectively for reads in Pool_R1_001.fasq.gz and in Pool_R2_001.fasq.gz). The parameter -c (— clean data, remove any read with an uncalled base) and -q (— discard reads with low quality scores) were not specified, being the data afterward processed with dDocent (http://ddocent.com/ Puritz et al. 2014a, b). Some dDocent steps accounted for base quality, needing a minimal trimming of the data. The used program “Trim Galore!” looked for double-digest RAD adapters and trimmed bases with quality scores less than PHRED 10*.* Low quality bases (below quality score of 20) were removed from the beginning and end of reads, and further assess an additional sliding 5 bp window that will trim bases when the average quality score drops below 10. Default setting for quality filter was used.

The demultiplexing of reads was performed with the process_radtags v. 1.42’for each sequencing lane separately, since the same adapter combinations were used among different libraries. Processed samples were renamed according to dDocent requirement (each individual was renamed following the scheme POP_ID, i.e. EATL_001). The output of 'process_radtags v. 1.42' for each sample consisted of four files: two files for correctly assigned sequences (pop_ID.1.fq.gz and pop_ID.2.fq.gz) and two for sequences discarded based on the quality filter settings (pop_ID.rem.1.fq.gz and pop_ID.rem.2.fq.gz).

Demultiplexed read files were renamed again according to dDocent requirement with the script available at <https://github.com/jpuritz/dDocent/raw/master/Rename_for_dDocent.sh> or alternatively with <rename -v "s/\.1\./\.F\./g" *> and <rename -v "s/\.2\./\.R\./g" *>. All discarded reads (rem files) were removed and the replicated individuals renamed to avoid conflicts or overwriting. All samples were merged in the same directory and samples with less than 500k reads were further removed from the dataset.

The resulting fastq files were checked with the FastQC software (www.bioinformatics.babraham.ac.uk/projects/fastqc/) that provides a report for Quality Check (QC) and highlights any potential bias in the raw data that may affect the downstream analysis, such a low-quality base calling (Del Fabbro et al. 2013).

The resulting dataset has been processed with dDocent v. 2.2.19 installed through MiniConda. The dDocent pipeline employs a series of data reduction techniques, alignment-based clustering, and, for PairedEnd assembly, a specialized RAD assembly software. This combination, according to the dDocent authors, allows for accurate and efficient de novo assembly increase accuracy with respect to the other available pipeline (i.e. STACKS and PyRAD, http://ddocent.com/why/). dDocent has been successfully employed in several recent works analyzing elasmobranch species (Dimens et al. 2019; Manuzzi et al. 2019; Barker et al. 2017; Portnoy et al. 2015) and in other marine fish species which are generally characterized by high diversity and low differentiation (Hollenbeck et al. 2017; Puritz et al. 2016).

To create a representative reference assembly, and to choose the optimal parameters in the construction of the reference assembly, at first, trimmed reads from 52 selected blue shark individuals (**Appendix 1**) were used to construct a reference assembly using the program CD-HIT (Fu et al. 2012). Individuals were selected according to the coverage within 0.5 standard deviations of the mean coverage of the total dataset, and representation of all sampling areas, size, and date of capture of the entire dataset, in order to avoid a site-based bias during the reference library construction, impacting downstream analysis.

In the reference assembly step, the custom bash scripts ReferenceOpt.sh and RefMapOpt.sh (<https://github.com/jpuritz/dDocent/tree/master/scripts>) were used in order to choose the best assembly parameters (cluster similarity, C; number of unique sequences with more than X coverage, counted within individuals, K1; number of unique sequences present in more than X Individuals, K2) for the reference assembly of the subset of 52 blue shark individuals.

After the identification of the optimal parameters (see Results Section), dDocent was then run again, and the program BWA (Li & Durbin, 2009) was used to map paired-end reads from all the blue shark individuals to the reference assembly using the matching score parameter (A), the mismatching score (B), and the gap penalty (O), set to 1, 3, and 5, respectively, which are the default values of BWA.

In order to minimize, an excess of homozygosity due to splitting alleles at a single locus into separate clusters, and inflated heterozygosity due to lumping multiple loci into a single contig, an haplotyping approach has been used (Willis et al. 2017). This approach also minimizes the effect of physical linkage of SNPs within a locus, that create artificial clustering in analyses that assumes markers are independent when a species with low levels of population divergence is investigated.

The same procedure was also detailed in a dedicated tutorial on the dDocent website (http://ddocent.com/filtering/). We applied several filtering steps to the raw SNPs dataset, according to dDocent filtering tutorial (<https://www.ddocent.com/filtering/>) and Puritz et al. (2016).

Then, after SNPs calling, starting from an initial Variant Calls file of 210 individuals and 56004 SNPs (TotalRawSNPs.vcf), we applied the following filtering steps:

**Filter#1**: all genotypes with less than five reads were converted into missing values

**Filter#2**: Removing all variants that are present below a minor allele frequency of 1% and not called in at least 50% of the samples.

**Filter#3**: use of the custom script [filter_missing_ind.sh](https://github.com/jpuritz/dDocent/blob/master/scripts/filter_missing_ind.sh) to filter out individuals with more than 60% of missing data.

**Filter#4**: use of a second custom bash script, called [pop_missing_filter.sh](https://github.com/jpuritz/dDocent/blob/master/scripts/pop_missing_filter.sh), for filtering loci that were not called in 75% of individuals in any one population, specified in the popmap.

**Filter#5**: filter sites again with minor allele frequency of 1%, and sites with less than 90% overall call rate.

**Filter#6**: Filtering use another custom script, called [dDocent_filters](https://github.com/jpuritz/dDocent/blob/master/scripts/dDocent_filters) (<https://github.com/jpuritz/dDocent/blob/master/scripts/dDocent_filters>) that use FreeBayes info criteria and depth. Specifically:

1. Loci with average allele balance at heterozygous genotypes less than 25% (the alternate allele in all heterozygous genotypes should have at least 25 or more reads). In addition, all loci with a quality sum of the reference or alternate allele equal to 0 were removed. This additionally filtering removes sites having large portions of spurious heterozygous genotype calls.
2. Loci with a quality score less than half of the total depth, since FreeBayes can inflate quality scores in case of excessive depth, were removed.
3. Loci with ratio between the mean mapping quality of alternate and reference allele less than 0.9 or more than 1.05 were removed.
4. Loci coming from the majority of reads that did not come from only one read orientation were removed, since the insert size is larger than paired-end read lengths, so true RAD loci should not have forward and reverse reads that overlap.
5. Loci were filtered on the base of properly paired-end reads status, since in the de novo assembly some loci will only have unpaired reads mapping to them. This is not a problem unless all the reads supporting the reference allele are paired but not support the alternate allele, suggesting a problem in the call. In fact, true variants, ideally, should have reads coming from all properly paired reads, or only from not properly paired reads (some RAD loci do not assemble well paired-end reads, leaving only the forward ones). Despite this, false variants tend to have properly paired reference reads and not properly paired alternate reads. Loci were retained if more than 0.05% of reference reads were properly paired and less than 0.05% of alternate reads were properly paired and vice versa.
6. Loci with a lower than the average depth plus on standard deviation were removed if the quality score was less than 2 times the depth. See Li (2014).
7. Only loci in the bottom 90% of mean depth were kept in order to remove any potential bias due to paralogs or repetitive regions in the genome (expected in sharks).

**Filter#7**: The resulting variant calls were decomposed into SNP and INDEL calls using vcflib, and INDELs were then removed using VCFtools.

**Filter#8:** The remaining SNPs were subsequently filtered on the base of the Hardy-Weinberg equilibrium per population, and the loci with a p-value less than 0,001 in at least the 50% of the populations were removed using the custom perl script filter_hwe_by_pop.pl (<https://github.com/jpuritz/dDocent/blob/master/scripts/filter_hwe_by_pop.pl)>.

**Filter#9:** Only biallelic SNPs were retained for further analysis

**Filter#10:** The resulting SNPs were transformed into haplotypes using the custom script [rad_haplotyper.pl](https://github.com/chollenbeck/rad_haplotyper) (<https://github.com/chollenbeck/rad_haplotyper>) from Willis et al. (2017), in order to mark any potential paralogs or genotype errors.

**Filter#11:** The output from Filter#10 was then use to create a list of files that had high levels of missing data and potential paralogs

**Filter#12:** Loci with more than 5 individuals marked as paralogous and more than 30 individuals marked for genotype errors were then removed from the dataset using the custom script [remove.bad.hap,loci.sh](https://github.com/jpuritz/dDocent/blob/master/scripts/remove.bad.hap.loci.sh) (<https://github.com/jpuritz/dDocent/blob/master/scripts/remove.bad.hap.loci.sh)>.

A relatedness analysis has been done with VCFtool, and then the replicas and the sibling individuals were removed.

A catalogue of SNPs loci with relative genotypes was produced at the end of the filtering procedure. From such a catalogue, genomic data were converted to the appropriate file format for subsequent genetic analysis with PDGSpider (Lischer and Excoffier 2012).

**Results**

From the sequencing of the 3 ddRAD libraries (Pg_ddRAD01, Pg_ddRAD02 and Pg_ddRAD03) a total of 1858 million of raw reads was obtained. After the demultiplexing and trimming, the number of retained reads per individual ranged from 21,183 to 66,488,950, with an average of 8,368,100 reads, and a total of 1807 million of reads. On the basis of the number of retained reads, all the samples of the Pg_ddRAD01 and Pg_ddRAD02 libraries were suitable for dDocent analysis. In the third library Pg_ddRAD_03, conversely, sequencing was not good for six individuals, with less than 500,000 retained reads (from 21,183 to 370,959 reads). Five of these individuals were archived blue shark specimens (collected in the period 2003 - 2005). Four individuals from the same period were instead suitable for analysis, with more than 1,350,000 retained reads.

Assessment of the outcomes of the sequencing for all the 3 ddRAD libraries was performed by FastQC, screening the quality of the retained reads for each sample, after demultiplexing and trimming. Outputs were:

- Acceptable for all Reads1 (R1, reads produced by the first sequencing reaction);
- Suboptimal for all Reads2 (R2, reads produced by the second sequencing reaction), in which Quality Value fell below 20 at the 124 or 129 base position, with similar patterns for all the libraries. Only in the first ddRAD blue shark library (Pg_ddRAD_01), the first 5 bases of all “Reverse” reads were to be eliminated.

Unfortunately, this is a common phenomenon for all 150 bp PE Illumina sequencing (i.e. the worsening of reads quality by increasing sequencing length).

After merging of all libraries files and removal of 6 samples with less than 500k reads (individuals WMED_073; EMED_050; EMED_047; EMED_042; EMED_049; EMED_048) the dataset consisted of 210 individuals (two files for each specimen, Reads1 and Reads2) including the 2 individuals EATL_015 and EMED_024 replicated in all 3 ddRAD blue shark Libraries, with the replica renamed to avoid conflicts or overwriting:

| Pg_ddRAD_02 | EATL_099 | EMED_099 |
| --- | --- | --- |
| Pg_ddRAD_03 | EATL_100 | EMED_100 |

Using data from selected 52 blue shark individuals (**Appendix 1**), the reference assembly with the best assembly parameters was constructed. The diagnostic script ReferenceOpt.sh identified the best cluster similarity threshold for the reference assembly at 0.9 (90%) testing different combinations of K1 and K2 (all possible combinations of K1-K2 from 1 to 17, **Figure S1**).

Then, testing again different K1 and K2 combinations, the diagnostic script RefMapOpt.sh, identified the best value for the number of unique sequences with more than X coverage (counted within individuals) and the best value for the number of unique sequences present in more than X individuals as K1=3 and K2=6, respectively (**Figure S2**). These K1-K2 cut-off values maximized the number of properly paired reads mapped and their coverage while minimizing the number of mismatched reads (improperly mapped).

These diagnostic scripts were extremely useful in the optimization of the parameters to be used in the construction of the reference library on which mapping the paired reads of all processed individuals and perform the SNPs calling. Since there is no reference genome for the blue shark, this reference library can be useful to map reads from RAD techniques carried out on Mediterranean and North Eastern Atlantic blue shark, since it was representative for the blue shark genetic variation in the target area.

The total raw dataset, derived from the read mapping and SNP calling steps using data from all processed individuals, consisted of 56,004 candidate loci.

Results obtained with the filtering process were:

**Filter#1** - all 56,004 genotypes were retained (all with at least 5 reads).

**Filter#2** - kept 27,863 out of a possible 56,004 sites after filtering for MAF<1% and filtering of the variants not called in at least 50% of samples.

**Filter#3** - 27,863 out of a possible 27,863 sites were retained after filter_missing_ind.sh filter.

**Filter#4** - 23,638 out of a possible 27,863 sites were retained after pop_missing_filter.sh, removing sites with more than 10% missing data in a single population.

**Filter#5** - after removing sites with MAF < 0.01, and sites with less than 90% overall call rate, 22,660 out of a possible 23,638 sites were retained.

**Filter#6** - after the dDocent_filters.sh 17,082 sites were retained.

The detailed results for filtering steps 1-6 were:

Number of sites filtered based on allele balance at heterozygous loci, locus quality, and mapping Quality / Depth: 2,535 of 22,660.

Number of additional sites filtered based on overlapping forward and reverse reads: 1,855 of 20,125.

Number of additional sites filtered based on properly paired status: 211 of 18,270.

Number of sites filtered based on high depth and quality score lower than 2*DEPTH: 976 of 18,059.

If distribution looks normal, a 1.645 sigma cut-off for mean depth (~90% of the data) would be 310,348.2065

Number of sites filtered based on maximum mean depth: 975 of 18,059. The maximum mean depth per site was set at 1,255 (**Figure S3**).

Total number of sites filtered: 5,578 of 22,660.

Remaining sites: 17,082.

**Filter#7** - after breaking complex mutational events (combinations of SNPs and INDELs) into separate SNP and INDEL calls, and then remove of the INDELs, 16,964 out of a possible 18,102 sites were identified.

**Filter#8** – Then, 92 loci were filtered out because not in HWE in more than half the populations using the custom script filter_hwe_by_pop.pl, leaving 16,872 of a possible 16,964 loci.

**Filter#9** -16,775 out of a possible 16,872 sites were retained, after restricting SNPs to loci with only 2 alleles.

**Filter#10** - after translating genotypes in haplotype, with rad_haplotyper.pl by Chris Hollenbeck from Willis et al. (2017), we had:

Rad_haplotyper filter output:

1. Filtered 1,635 loci below missing data cut-off.
2. Filtered 192 possible paralogs.
3. Filtered 0 loci with low coverage or genotyping errors.
4. Filtered 0 loci with an excess of haplotypes.

**Filter#11** - The resulting output has been moved and a list of files that had high levels of missing data and potential paralogs has been created.

**Filter#12** – Loci with more than 5 individuals marked as paralogous and more than 30 individuals marked for genotype errors were then removed from the dataset using the custom script remove.bad.hap,loci.sh, resulting in a final dataset of 14729 sites

Commented filtering steps can be found in **Appendix 2**

Only the replicated samples with the lowest number of missing values (NA) were kept for further analyses. Samples with more than 20% of missing values were also discarded. Only one samples with more than 20% of NA was removed (EMED_051 NA=22.81%)

Comparing replicated samples, samples EATL_15 and EMED_100 were selected, the other removed.

In the subsequent analyses, the sample EMED_100 were renamed as EMED_024.

| Replica1 | NA | Replica2 | NA |
| --- | --- | --- | --- |
| EATL_15 | 0.44% | EMED_024 | 0.44% |
| EATL_99 | 0.44% | EMED_099 | 0% |
| EATL_100 | 0.53% | EMED_100 | 0% |

When assessing relatedness, we found two pairs of unintentional duplicates, and for each pair we discarded the one with the highest amount of NA %.

| Pair | INDV1 | INDV2 | RELATEDNESS |
| --- | --- | --- | --- |
| 1 | NEATL_001 | NEATL_005 | 1.0469 |
| 2 | NEATL_003 | NEATL_007 | 0.866365 |

| Pair1 | NA | Pair2 | NA |
| --- | --- | --- | --- |
| NEATL_001 | 0.35% | NEATL_003 | 0.35% |
| NEATL_005 | 0.44% | NEATL_007 | 0.27% |

NEATL_001 and NEATL_007 were retained in the dataset.

In total, seven individuals were removed from the dataset (EMED_051; EATL_099; EATL_100; EMED_024; EMED_099; NEATL_005; NEATL_003).

The resulting dataset consisted of 14,729 SNPs and 203 blue shark individuals.

**Supplementary Text: Outlier SNPs detection**

In Bayescan, 20 pilot runs of 5000 iterations each were run with a burn-in of 50 000 iterations and 5000 samples with a thinning interval of 10 and a prior odd of 10. The q-value of a given locus is the Minimum False Discovery Rate (FDR) at which this locus may become significant, where the FDR is the expected proportion of false positives among outlier markers, while the alpha value is defined as the locus-population F_ST_ coefficient shared by all the populations using a logistic regression (see Bayescan manual) Loci with an alpha value significantly > 0 (possibly indicative of diversifying selection) and q-values < 0.05 were considered “outliers” following the software manual.

*Pcadapt* uses   Principal Component Analysis to select potential outlier loci. The proportion of variance explained by each PC was estimated with the "screeplot function" and the PCA structure using the "score plot". For a given SNP, the test statistic is based on the z-scores obtained when regressing SNPs with the K principal components. The test statistic for detecting outlier SNPs is the Mahalanobis distance (Mahalanobis, 1936), which is a multi-dimensional approach that measures how distant is a point. The number of Principal Components selected as the best for the dataset, looking at the percentage of variance explained by each PC in a Scree Plot (as suggested by the authors of the method), was K = 2.

OutFLANK is an R package implementing the method developed by Whitlock and Lotterhos (2015), based on fitting the empirical distribution of F_ST_ values at neutral loci to a chi-square distribution, trimming high and low F_ST_ values considered potentially under diversifying or balancing selection. Then, the distribution is compared to the empirical data, assigning q-values to each locus, identifying the outliers as those loci outside the expected distribution. A set of random and quasi-independent SNPs was used to calculate mean F_ST_ and the degrees of freedom on the chi-square distribution. In order to minimize false positives rate, potential Linkage Disequilibrium event has been investigated visually along the SNP dataset using a loading plot for each PC (**Figure S4**).

**Supplementary Text: Population genetics analysis**

The population genetics analysis made in the main manuscript, has been done also on nine subgroups as showed in the **Figure S7**: CELT, Celtic Sea; EATL, North Eastern Atlantic; BALE, Balearic Sea; LIGU, Ligurian Sea; TYRR, Tyrrhenian Sea; WION, Western Ionian Sea; EION, Eastern Ionian Sea; AEGE, Aegean and Levantine Sea (South of Crete); ADRI, Adriatic Sea.

After removing the five most divergent eastern Mediterranean samples; 3 from the Adriatic Sea (EMED_020, EMED_032, EMED_053), one from the eastern Ionian Sea (EMED_011), and one from Crete (EMED_052), we filtered the dataset as explained in the Rmarkdown Appendix S3, creating a dataset of 198 specimens and 14’627 loci. The genetic diversity and the genetic differentiation (F_ST_) estimated, is totally comparable to those estimations observed using the totality of samples (including the most divergent). The only difference is a lower value of F_ST_ when comparing the Eastern Mediterranean with the Western Mediterranean, which is still significant.

For the Mantel test for isolation by distance per population, the points were chosen in order to represent the marine distance among areas accurately. When two groups within the same area (e.g., Balearic Sea vs Ligurian Sea vs South Tyrrhenian Sea in the WMED) were distant to each other, a midpoint was chosen as representative of the entire area (WMED) using the centroid formula and accounting for one sample for each area. The case of the EMED is particular because a centroid point between the Adriatic Sea and the Levantine Sea would significantly decrease the real distance between the sharks within the EMED and the sharks within the WMED, creating a bias. In this case, we left the South of Crete as a representative point, since the distance between the South of Crete and the WMED is comparable to the distance between the sharks from the Central Adriatic Sea and the WMED. Detailed analytical steps are showed in the Rmarkdown in Appendix 3.Commented analysis steps can be found in rmarkdown document in **Appendix 3**.

**SUPPORTING REFERENCES**

Barker, A.M., Frazier, B.S., Bethea, D.M., Gold, J.R., Portnoy, D.S. (2017). Identification of young-of-the-year great hammerhead shark *Sphyrna mokarran* in northern Florida and South Carolina. *Journal of Fish Biology*, 91(2):664-668. doi:10.1111/jfb.13356.

Catchen, J., Hohenlohe, P.A., Bassham, S., Amores, A., Cresko, W.A. (2013). Stacks: an analysis tool set for population genomics. *Molecular Ecology*, 22(11):3124-3240. doi: https://doi.org/10.1111/mec.12354.

Catchen, J.M., Amores, A., Hohenlohe, P., Cresko, W., Postlethwait, J.H. (2011). Stacks: building and genotyping loci de novo from short-read sequences. *G3: Genes, Genomes, Genetics*, 1(3):171-82. doi: 10.1534/g3.111.000240.

Del Fabbro, C., Scalabrin, S., Morgante, M., Giorgi, F.M. (2013) An Extensive Evaluation of Read Trimming Effects on Illumina NGS Data Analysis. *PLoS ONE*, 8(12): e85024. doi: <https://doi.org/10.1371/journal.pone.0085024>.

Dimens, P.V., Willis, S., Grubbs R.D., Portnoy, D.S. (2019). A genomic assessment of movement and gene flow around the South Florida vicariance zone in the migratory coastal blacknose shark, *Carcharhinus acronotus*. *Marine Biology*, 166:86. <https://doi.org/10.1007/s00227-019-3533-1>

Fu, L., Niu, B., Zhu, Z., Wu, S., Li, W. (2012). CD-HIT: Accelerated for clustering the next generation sequencing data. *Bioinformatics*, 28(23):3150–3152. doi: 10.1093/bioinformatics/bts565.

Hollenbeck, C.M., Portnoy, D.S., Wetzel, D., Sherwood, T.A., Samollow, P.B., Gold, J.R. (2017). Linkage Mapping and Comparative Genomics of Red Drum (*Sciaenops ocellatus*) Using Next-Generation Sequencing. G3, 7(3):843-850. doi: 10.1534/g3.116.036350.

Li, H. (2014). Toward better understanding of artifacts in variant calling from high-coverage samples. *Bioinformatics,* 30, 2843-2851. (doi:10.1093/bioinformatics/btu356)

Li, H., Durbin, R. (2009). Fast and accurate short read alignment with Burrows-Wheeler transform. *Bioinformatics*, 25(14):1754-1760. doi: 10.1093/bioinformatics/btp324.

Mahalanobis, P.C. (1936). On the generalized distance in statistics. *Proceedings of the National Institute of Science of India* 2(1): 49-55.

Manuzzi, A., Zane, L., Muñoz-Merida, A., Griffiths, A.M., Veríssimo, A. (2019). Population genomics and phylogeography of a benthic coastal shark (Scyliorhinus canicula) using 2b-RAD single nucleotide polymorphisms. Biological Journal of the Linnean Society, 126(2): 289–303. doi: 10.1093/biolinnean/bly185

Portnoy, D.S., Puritz, J.B., Hollenbeck, C.M., Gelsleichter, J., Chapman, D., Gold, J.R. (2015) Selection and sex-biased dispersal in a coastal shark: the influence of philopatry on adaptive variation. Molecular Ecology, 24(23):5877-5885. doi: 10.1111/mec.13441.

Puritz, J.B., Gold, J.R., Portnoy, D.S. (2016). Fine-scale partitioning of genomic variation among recruits in an exploited fishery: causes and consequences. *Scientific Reports*, 6:36095. doi: doi:10.1038/srep36095.

Puritz, J.B., Matz, M.V., Toonen, R.J., Weber, J.N., Bolnick, D.I. (2014a). Demystifying the RAD fad. *Molecular Ecology*, 23(24):5937-5942. doi: 10.1111/mec.12965.

Puritz, J.B., Hollenbeck, C.M., Gold, J.R. (2014b). dDocent: a RADseq, variant-calling pipeline designed for population genomics of non-model organisms. *PeerJ*, 10(2): e431. doi: 10.7717/peerj.431.

Willis, S.C., Hollenbeck, C.M., Puritz, J.B., Gold, J.R., Portnoy, D.S. (2017). Haplotyping RAD loci: an efficient method to filter paralogs and account for physical linkage. *Molecular Ecology Resources*, 17(5):955-965. doi: 10.1111/1755-0998.12647.

Whitlock, M.C. & Lotterhos, K.E. (2015). Reliable Detection of Loci Responsible for Local Adaptation: Inference of a Null Model through Trimming the Distribution of F_ST_. *The American Naturalist*, 186, S24–S36. doi: https://doi.org/10.1086/682949

**Supplementary Tables**

**Table S1** *The MEDBLUESGEN database content. List of blue sharks collected with associated biological and fishery data. All data is reported.*

**Table S2** *Geographical composition of the* *three ddRAD libraries of blue shark*

| **ddRAD library** | **Area** | | | | **Total** |
| --- | --- | --- | --- | --- | --- |
|  | **CELT** | **EATL** | **WMED** | **EMED** |  |
| Pg_ddRAD_01 |  | 28 | 15 | 29 | **72** |
| Pg_ddRAD_02 | 20 | 5 | 33 | 12 | **70** |
| Pg_ddRAD_03 | 10 |  | 41 | 19 | **70** |
| **Total** | **30** | **33** | **89** | **60** | **212** |

**Table S3** *Temporal and geographical composition of the blue shark dataset after filtering of ddRAD sequencing data.*

| **AREA** | **UNKNOWN** | **2003** | **2004** | **2008** | **2009** | **2012** | **2013** | **2014** | **2015** | **2016** | **Total** |
| --- | --- | --- | --- | --- | --- | --- | --- | --- | --- | --- | --- |
| **CELT** | **28** |  |  |  |  |  |  |  |  |  | **28** |
| **EATL** |  |  |  |  |  |  |  | **9** | **24** |  | **33** |
| **EMED** |  | **2** | **3** |  |  | **1** |  | **3** | **45** |  | **54** |
| **WMED** |  |  |  | **1** | **1** | **4** | **1** | **53** | **15** | **13** | **88** |
| **Total** | **28** | **2** | **3** | **1** | **1** | **5** | **1** | **65** | **84** | **13** | **203** |

**Table S4:** *Results from mapping the flanking region of the outlier SNPs against the GenBank database. Only the match with the lowest E-value is shown.*

| **SNP** | **Definition** | **E-value** | **Accession** |
| --- | --- | --- | --- |
| dDocent_Contig_1981_250 | No significant similarity found |  |  |
| dDocent_Contig_2220_275 | No significant similarity found |  |  |
| dDocent_Contig_2224_53 | Phlogophora meticulosa genome assembly, chromosome: 24 | 1.70E-02 | LR990541.1 |
| dDocent_Contig_2246_20 | *Scyliorhinus canicula* chromosome 20 | 6.00E-07 | LR744049.1 |
| dDocent_Contig_2474_64 | No significant similarity found |  |  |
| dDocent_Contig_2514_164 | *Scyliorhinus canicula* chromosome 21 | 5.00E-47 | LR744050.1 |
| dDocent_Contig_2554_27 | *Scyliorhinus canicula* chromosome 20 | 3.00E-14 | LR744049.1 |
| dDocent_Contig_2761_197 | *Triakis scyllium* IL-1 gene for interleukin-1beta, complete cds | 8.00E-40 | AB074142.1 |
| dDocent_Contig_3610_282 | No significant similarity found |  |  |
| dDocent_Contig_3642_75 | PREDICTED: *Carcharodon carcharia*s collagen alpha-6(VI) chain-like (LOC121276073), mRNA | 1.00E-06 | XM_041184013.1 |
| dDocent_Contig_3709_163 | No significant similarity found |  |  |
| dDocent_Contig_3874_14 | PREDICTED: *Pristis pectinata* uncharacterized LOC127577890 (LOC127577890), mRNA | 1.00E-11 | XM_052029530.1 |
| dDocent_Contig_4280_38 | *Heterodontus francisci* Evx2 (evx2), HoxD14 (HoxD14), HoxD13 (HoxD13), HoxD12 (HoxD12), HoxD11 (HoxD11), HoxD10 (HoxD10), HoxD9 (HoxD9), HoxD8 (HoxD8), HoxD5 (HoxD5), HoxD4 (HoxD4), HoxD3 (HoxD3), HoxD2 (HoxD2), and HoxD1 (HoxD1) genes, complete cds | 4.00E-50 | AF224263.2 |
| dDocent_Contig_4321_101 | *Scyliorhinus canicula* chromosome 20 | S7e-34 | LR744049.1 |
| dDocent_Contig_4466_47 | *Scyliorhinus canicula* chromosome 25 | 1.00E-41 | LR744054.1 |
| dDocent_Contig_4469_118 | *Scyliorhinus canicula* chromosome 20 | 2.00E-15 | LR744049.1 |

**Table S5:** Genetic diversity estimates per geographic areas - Allelic richness (Ar) with the low and high CI, Number of individuals (Nb), Observed heterozygosity (Hobs), Expected heterozygosity (Hexp), Unbiased Expected heterozygosity (Hexp_un), inbreeding coefficient (Fis) with the low and high CI on Fis wrapper, *p*-values from chi-square test for goodness-of-fit to Hardy-Weinberg equilibrium (HWE), test significance for directional HWE on homozygote and heterozygote deficiency (hwe_hom; hwe_het). Abbreviations: CELT, Celtic Sea; EATL, North eastern Atlantic; BALE, Balearic Sea; LIGU, Ligurian Sea; TYRR, Tyrrhenian Sea; WION, western Ionian Sea; EION, eastern Ionian Sea; AEGE, Aegean and Levantine Sea (South of Crete); ADRI, Adriatic Sea.

|  | Ar | Nb | Hobs | Hexp | Hexp_un | Fis | hwe_glb | hwe_hom | hwe_het |
| --- | --- | --- | --- | --- | --- | --- | --- | --- | --- |
| CELT | 1.564 (1.488 - 1.598) | 28 | 0.151 | 0.163 | 0.166 | 0.054 (0.028 - 0.046) | 1 | 1 | 1 |
| EATL | 1.574 (1.528 - 1.602) | 33 | 0.151 | 0.163 | 0.166 | 0.059 (0.037 - 0.051) | 1 | 1 | 1 |
| BALE | 1.575 (1.525 - 1.601) | 41 | 0.15 | 0.164 | 0.166 | 0.068 (0.047 - 0.062) | 1 | 1 | 1 |
| LIGU | 1.578 1.528 - 1.608) | 39 | 0.153 | 0.165 | 0.167 | 0.059 (0.039 - 0.051) | 1 | 1 | 1 |
| TYRR | 1.507 (1.443 - 1.573) | 8 | 0.154 | 0.157 | 0.167 | 0 (-0.101 - -0.012) | 1 | 1 | 1 |
| WION | 1.543 (1.487 - 1.599) | 15 | 0.153 | 0.16 | 0.166 | 0.029 (-0.023 - 0.019) | 1 | 1 | 1 |
| EION | 1.511 (1.442 - 1.573) | 9 | 0.152 | 0.157 | 0.166 | 0.008 (-0.087 - -0.003) | 1 | 1 | 1 |
| AEGE | 1.536 (1.439 - 1.604) | 9 | 0.172 | 0.165 | 0.175 | -0.047 (-0.186 - -0.026) | 1 | 1 | 1 |
| ADRI | 1.565 (1.495 - 1.613) | 21 | 0.159 | 0.165 | 0.169 | 0.022 (-0.023 - 0.023) | 1 | 1 | 1 |

**Table S6** *Pairwise* F_ST_ *values (below diagonal) and associated p-values (above diagonal) between blue shark samples based on the 14713 neutral SNPs. Values in bold significant after false discovery correction for multiple tests.* Abbreviations: CELT, Celtic Sea; EATL, North eastern Atlantic; BALE, Balearic Sea; LIGU, Ligurian Sea; TYRR, Tyrrhenian Sea; WION, western Ionian Sea; EION, eastern Ionian Sea; AEGE, Aegean and Levantine Sea (South of Crete); ADRI, Adriatic Sea.

|  | CELT | EATL | BALE | LIGU | TYRR | WION | EION | AEGE | ADRI |
| --- | --- | --- | --- | --- | --- | --- | --- | --- | --- |
| CELT | \ | 0.1285 | 0.1699 | 0.0907 | 0.7463 | **0.0028** | **0.0037** | 0.6888 | **0** |
| EATL | 0.000351 | \ | 0.0252 | 0.092 | 0.5579 | 0.1803 | **0.0017** | 0.8548 | **0** |
| BALE | 0.000257 | 0.000476 | \ | 0.5298 | 0.9118 | 0.8814 | **0.0019** | 0.9847 | **0** |
| LIGU | 0.000389 | 0.000344 | -7.75E-06 | \ | 0.9665 | 0.6562 | 0.1203 | 0.9337 | **0** |
| TYRR | -0.00048 | -0.00011 | -8.86E-04 | -0.00117 | \ | 0.7942 | 0.9702 | 0.9978 | **0.0149** |
| WION | **0.001277** | 0.00039 | -0.00045 | -0.00016 | -0.0007 | \ | 0.2723 | 0.2772 | 0.2008 |
| EION | **0.001822** | **0.001993** | **0.001938** | 0.000739 | -0.00195 | 0.000472 | \ | 1 | **0.0049** |
| AEGE | -0.0003 | -0.00062 | -0.00131 | -0.00088 | -0.00279 | 0.000459 | -0.00423 | \ | 0.3567 |
| ADRI | **0.002794** | **0.002862** | **0.002082** | **0.00147** | **0.001724** | 0.000393 | **0.00188** | 0.000265 | \ |

**Table S7:** *Genetic diversity estimates per geographic areas using 14627 SNPs after removing highly diverged samples XXX - Allelic richness (Ar) with the low and high CI, Number of individuals (Nb), Observed heterozygosity (Hobs), Expected heterozygosity (Hexp), Unbiased Expected heterozygosity (Hexp_un), inbreeding coefficient (Fis) with the low and high CI on Fis wrapper, p-values from chi-square test for goodness-of-fit to Hardy-Weinberg equilibrium (HWE), test significance for directional HWE on homozygote and heterozygote deficiency (hwe_hom; hwe_het). Abbreviations: CELT, Celtic Sea; EATL, North eastern Atlantic; WMED, western Mediterranean; EMED, eastern Mediterranean.*

|  | Ar | | Nb | Hobs | Hexp | Hexp_un | Fis | hwe_glb | hwe_hom | hwe_het |
| --- | --- | --- | --- | --- | --- | --- | --- | --- | --- | --- |
| CELT | 1.767 (1.727 - 1.799) | 28 | | 0.151 | 0.163 | 0.166 | 0.054 (0.028 - 0.046) | 1 | 1 | 1 |
| EATL | 1.784 (1.747 - 1.816) | 33 | | 0.151 | 0.163 | 0.166 | 0.059 (0.038 - 0.051) | 1 | 1 | 1 |
| WMED | 1.834 (1.804 - 1.857) | 88 | | 0.152 | 0.166 | 0.167 | 0.077 (0.064 - 0.072) | 0 | 1 | 0 |
| EMED | 1.810 (1.777 - 1.838) | 48 | | 0.159 | 0.167 | 0.169 | 0.048 (0.020 - 0.051) | 1 | 1 | 1 |

**Table S6** *Pairwise* F_ST_ *values (below diagonal) and associated p-values (above diagonal) between blue shark samples based on the 14627 neutral SNPs. Values in bold significant after false discovery correction for multiple tests.* Abbreviations: CELT, Celtic Sea; EATL, North eastern Atlantic; WMED, western Mediterranean; EMED, eastern Mediterranean.

|  | CELT | EATL | WMED | EMED |
| --- | --- | --- | --- | --- |
| CELT | \ | 0.1093 | 0.0830 | **0** |
| EATL | 0.00038 | \ | **0.0083** | **0** |
| WMED | 0.00031 | **0.00044** | \ | **0.0201** |
| EMED | **0.00135** | **0.00122** | **0.00028** | \ |

**Supplementary Figure**

**Figure S1:** *Best cluster similarity value, C, identified using the diagnostic script ReferenceOpt.sh and 17 combinations of K1 and K2. The best threshold is identified at the point of inflection on the curve*

**
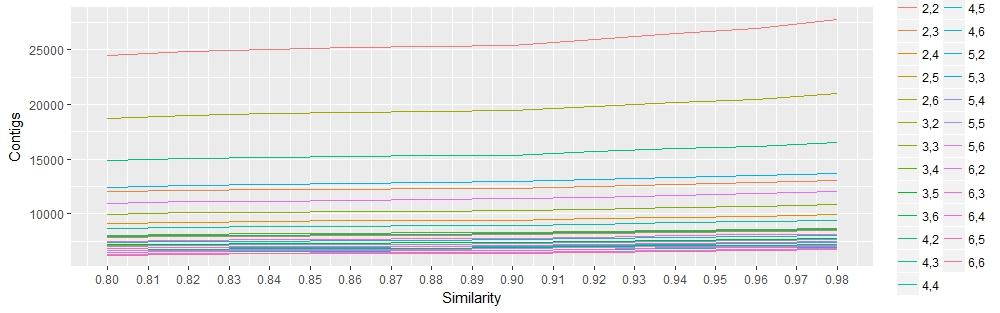
**

**Figure S2:** *Best K1K2 combination identified using the diagnostic script RefMapOpt.sh***
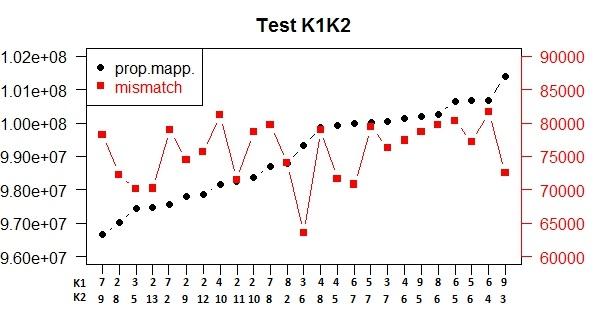
**

**Figure S3:** *Plot of the number of variable sites (y-axis) and mean depth per site (x-axis).*


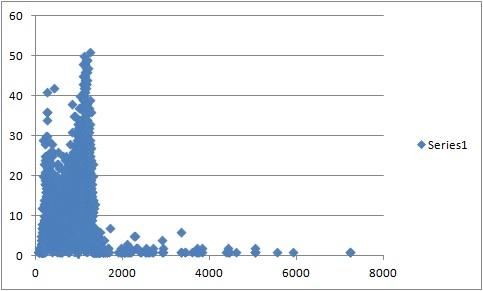


**Figure S4:** *PCA-related loading plots (contributions of each SNP to the PC) showing that PCs are not determined by a single genomic region, which is likely to be free of strong Linkage Disequilibrium.*

**
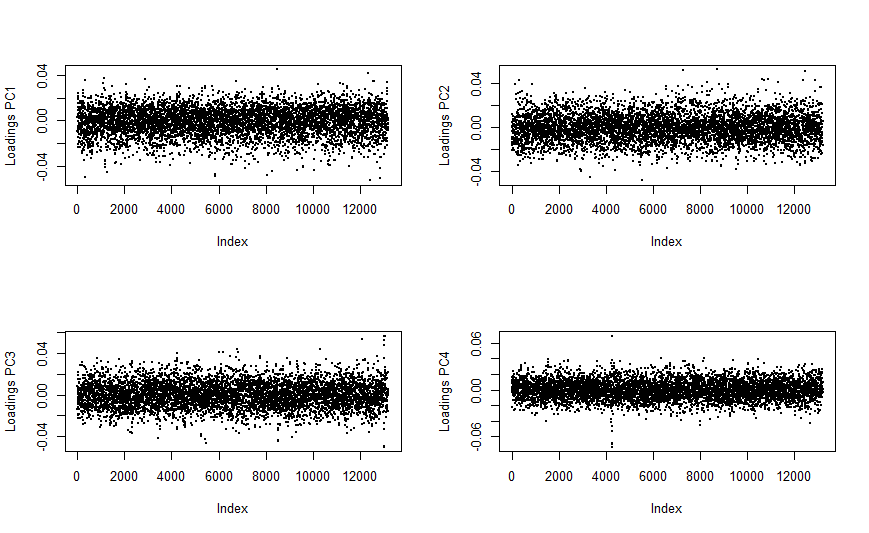
**

**Figure S5:** *Least cost distances between the four main geographical areas of each subpopulation by seaway. CELT: Celtic Sea, EATL: North eastern Atlantic, WMED: western Mediterranean, EMED: eastern Mediterranean. Least cost distance in km, CELT-EATL: 1927*


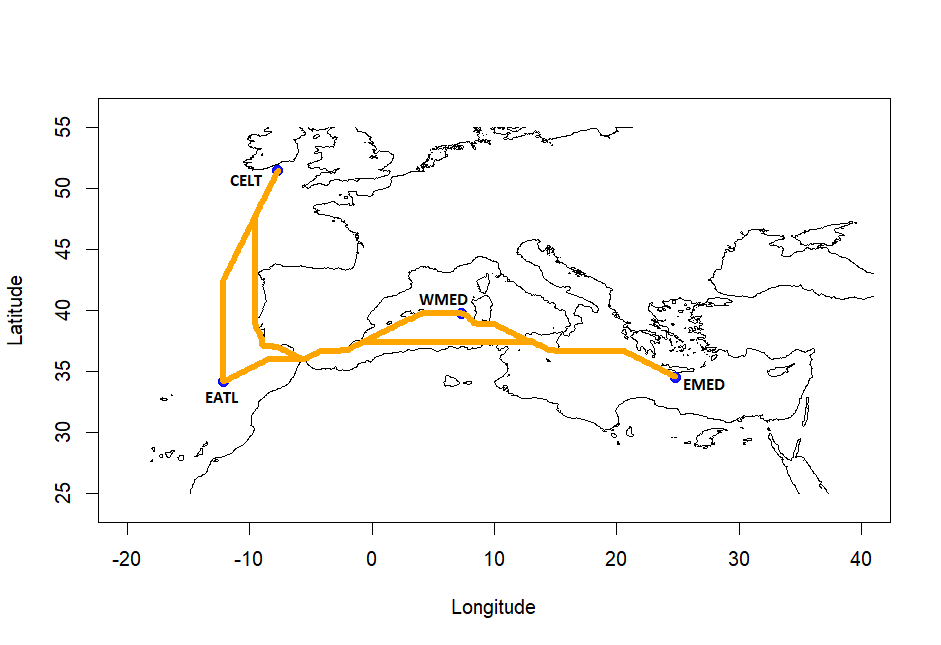


**Figure S6:** *Isolation by distance plot with relative Mantel statistics. Dgen: genetic distance in pairwise* F_ST_*, Dgeo: geographical distance in km. Each point represents a comparison between two subpopulations as designed by colours.*


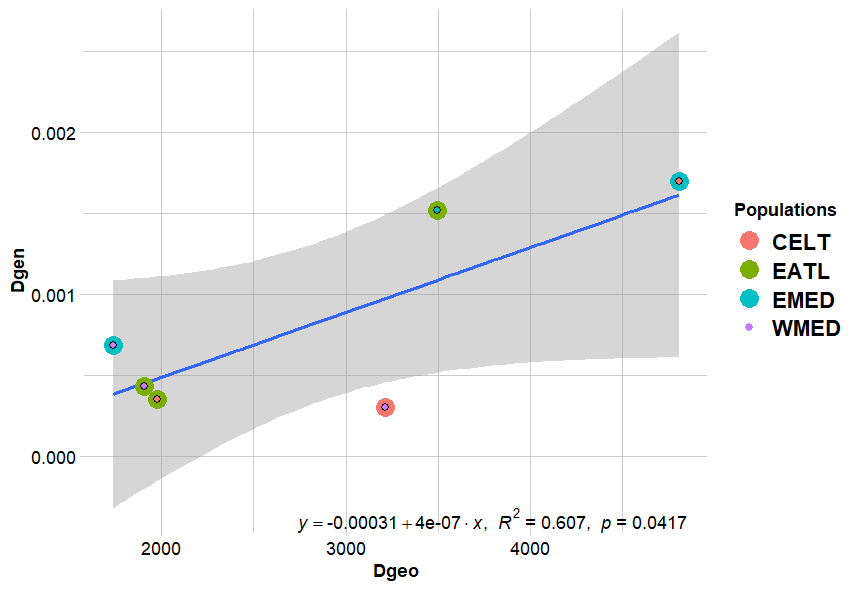


**Figure S7:** *Maps of sampling locations of blue shark in the Celtic Sea (green dots), North Eastern Atlantic (red dots), Western Mediterranean (purple dots) and Eastern Mediterranean (blue dots). The orange dots represent the genetically most divergent Eastern Mediterranean blue shark, in the Adriatic Sea, in the Ionian Sea, and in the North of Crete. Blue shading indicates bathymetry (i.e, depth, in meters).* Abbreviations: CELT, Celtic Sea; EATL, North Eastern Atlantic; BALE, Balearic Sea; LIGU, Ligurian Sea; TYRR, Tyrrhenian Sea; WION, Western Ionian Sea; EION, Eastern Ionian Sea; CRET, Sea of Crete, and South of Crete, including Lybian Sea; ADRI, Adriatic Sea.

**
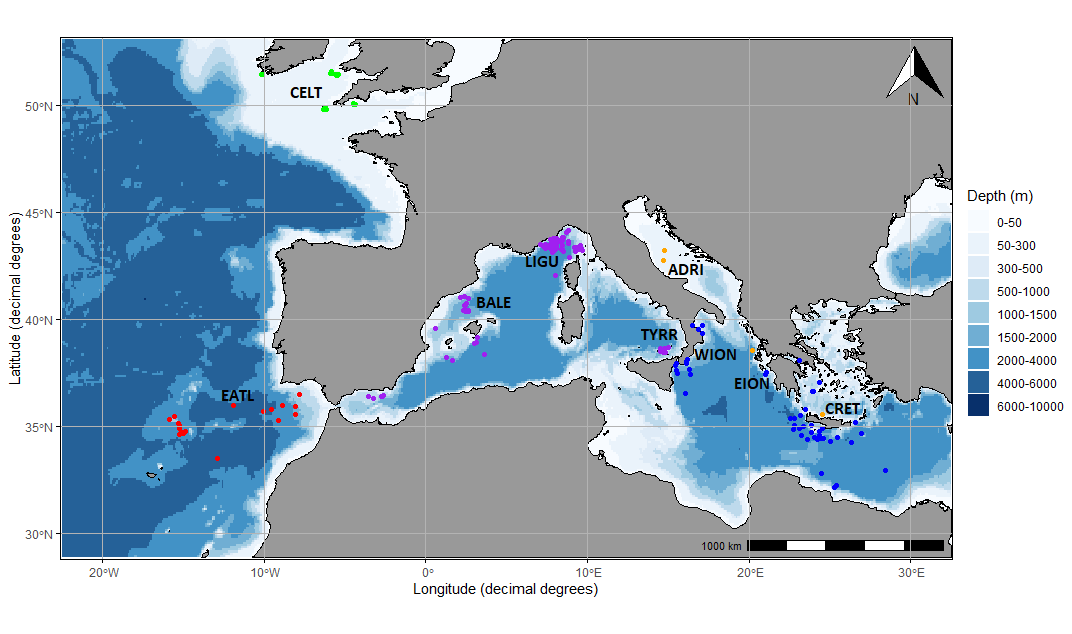
**

**Figure S8:** *Analysis of the Principal Components (PCA) plot using 14,713 neutral SNPs dataset on nine subdivided groups. Abbreviations: CELT, Celtic Sea; EATL, North Eastern Atlantic; BALE, Balearic Sea; LIGU, Ligurian Sea; TYRR, Tyrrhenian Sea; WION, Western Ionian Sea; EION, Eastern Ionian Sea; CRET, Sea of Crete (Aegean Sea) and South of Crete (Libyan Sea); ADRI, Adriatic Sea.*


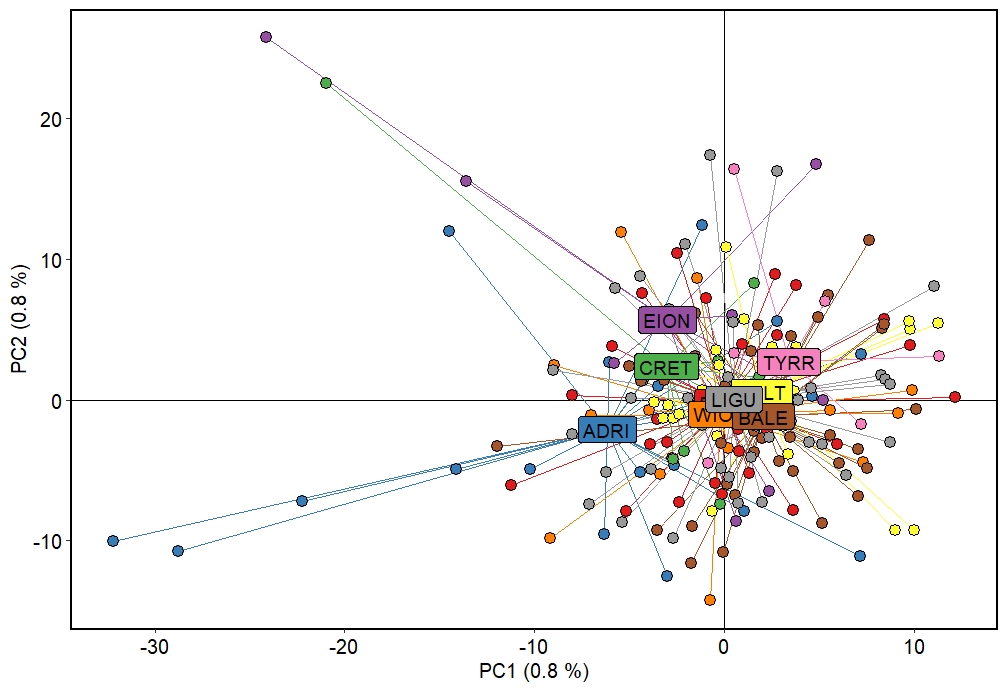


**Figure S9:** *Discriminant Analysis of the Principal Components (DAPC) plot using 14,713 neutral SNPs dataset on nine subdivided groups. Abbreviations: CELT, Celtic Sea; EATL, North Eastern Atlantic; BALE, Balearic Sea; LIGU, Ligurian Sea; TYRR, Tyrrhenian Sea; WION, Western Ionian Sea; EION, Eastern Ionian Sea; CRET, Sea of Crete (Aegean Sea) and South of Crete (Libyan Sea); ADRI, Adriatic Sea.*

**
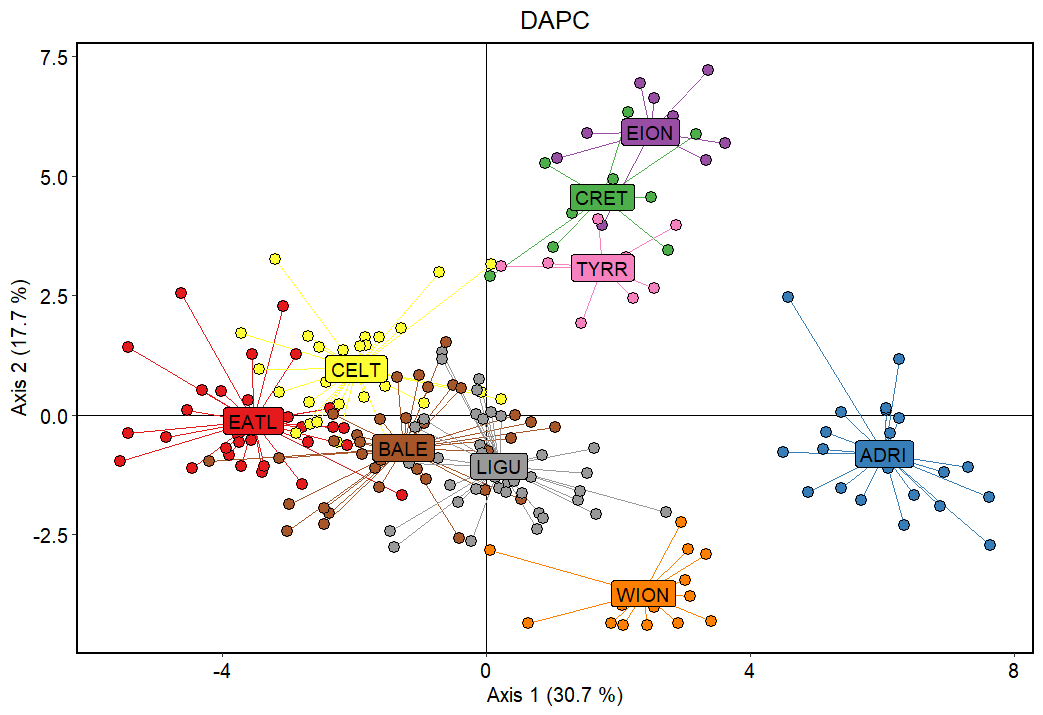
**

**Appendix 1**

List of samples selected as representatives of the total variation in the dataset. AREAS: CELT: Celtic Sea; EATL: North Eastern Atlantic; BALE: Balearic Sea; LIGU: Ligurian Sea; TYRR: Tyrrhenian Sea; IONI: Ionian Sea; AEGE: Aegean Sea; ADRI: Adriatic Sea.

| **MEDBLUESGEN CODE** | **Filename** | **AREA** | **Reads** |
| --- | --- | --- | --- |
| BSH_EATL_IEO_2014_J_024 | EATL_002 | EATL | 2202294 |
| BSH_EATLA_IEO_15_J_057 | EATL_012 | EATL | 2287612 |
| BSH_EATLA_IEO_15_J_063 | EATL_015 | EATL | 3056520 |
| BSH_EATLA_IEO_15_L_053 | EATL_025 | EATL | 3764378 |
| BSH_EATLA_IEO_15_L_054 | EATL_026 | EATL | 3290910 |
| BSH_EATLA_IEO_14_J_023 | EATL_010 | EATL | 4566158 |
| BSH_EMED_NKUA_2003_J_038 | EMED_030 | AEGE | 1189129 |
| BSH_EMED_NKUA_2004_L_011 | EMED_045 | AEGE | 1324312 |
| BSH_EMED_NKUA_15_J_001 | EMED_004 | AEGE | 2260818 |
| BSH_EMED_NKUA_15_J_002 | EMED_005 | AEGE | 4181482 |
| BSH_EMED_NKUA_15_J_014 | EMED_007 | AEGE | 3279191 |
| BSH_EMED_NKUA_15_L_007 | EMED_010 | AEGE | 4377882 |
| BSH_EMED_NKUA_2015_L_009 | EMED_052 | AEGE | 4504215 |
| BSH_EMED_NKUA_15_L_010 | EMED_012 | AEGE | 4882867 |
| BSH_EMED_UNIBO_15_J_005 | EMED_018 | ADRI | 2739238 |
| BSH_EMED_UNIBO_15_J_010 | EMED_023 | ADRI | 3844219 |
| BSH_EMED_UNIBO_15_J_012 | EMED_025 | ADRI | 3489608 |
| BSH_EMED_UNIBO_15_J_013 | EMED_026 | ADRI | 3425321 |
| BSH_EMED_UNIBO_2015_J_011 | EMED_099 | ADRI | 4679476 |
| BSH_EMED_UNIBO_15_J_007 | EMED_020 | ADRI | 3507540 |
| BSH_EMED_UNICAL_2014_J_022 | EMED_002 | IONI | 1966252 |
| BSH_EMED_UNICAL_2014_J_023 | EMED_036 | IONI | 4680418 |
| BSH_EMED_UNICAL_2014_L_025 | EMED_003 | IONI | 2034179 |
| BSH_EMED_UNICAL_2015_J_014 | EMED_040 | IONI | 3355180 |
| BSH_EMED_UNICAL_2015_J_018 | EMED_058 | IONI | 1721091 |
| BSH_EMED_UNICAL_2015_J_015 | EMED_055 | IONI | 3326261 |
| BSH_EATL_QUB_2007_L_003 | NATL_003 | CELT | 1874876 |
| BSH_EATL_QUB_2007_L_004 | NATL_004 | CELT | 1969517 |
| BSH_EATL_QUB_2007_L_022 | NATL_022 | CELT | 1956903 |
| BSH_EATL_QUB_2007_L_006 | NATL_006 | CELT | 1975839 |
| BSH_EATL_QUB_2007_L_028 | NATL_028 | CELT | 2021651 |
| BSH_EATL_QUB_2007_L_008 | NATL_008 | CELT | 1801276 |
| BSH_EATL_QUB_2007_L_029 | NATL_029 | CELT | 2259362 |
| BSH_WMED_IEO_2014_J_029 | WMED_022 | BALE | 2621086 |
| BSH_WMED_IEO_2014_J_040 | WMED_026 | BALE | 3557127 |
| BSH_WMED_IEO_2014_L_009 | WMED_058 | BALE | 3509306 |
| BSH_WMED_IEO_2014_L_035 | WMED_064 | BALE | 2615914 |
| BSH_WMED_IEO_2014_L_037 | WMED_066 | BALE | 2652290 |
| BSH_WMED_IEO_2014_J_039 | WMED_025 | BALE | 3332249 |
| BSH_WMED_UNICAL_15_J_002 | WMED_005 | TYRR | 2366209 |
| BSH_WMED_UNICAL_15_J_005 | WMED_007 | TYRR | 4843687 |
| BSH_WMED_UNICAL_2015_J_003 | WMED_030 | TYRR | 3762281 |
| BSH_WMED_UNICAL_2015_J_006 | WMED_031 | TYRR | 3472639 |
| BSH_WMED_UNICAL_2015_J_009 | WMED_074 | TYRR | 3321733 |
| BSH_WMED_UNICAL_2015_J_010 | WMED_075 | TYRR | 5041349 |
| BSH_WMED_UNIGE_15_J_005 | WMED_013 | LIGU | 2504439 |
| BSH_WMED_UNIGE_2008_J_047 | WMED_033 | LIGU | 2750852 |
| BSH_WMED_UNIGE_2012_L_026 | WMED_076 | LIGU | 2502172 |
| BSH_WMED_UNIGE_2014_J_019 | WMED_080 | LIGU | 2081629 |
| BSH_WMED_UNIGE_2016_J_060 | WMED_085 | LIGU | 2063341 |
| BSH_WMED_UNIGE_2014_J_015 | WMED_038 | LIGU | 3090239 |
| BSH_WMED_UNIGE_15_J_001 | WMED_012 | LIGU | 2935667 |
