# Supplementary material for: Population Genomics of the Blue Shark, Prionace glauca, Reveals Different Populations in the Mediterranean Sea and the Northeast Atlantic
Source: Evol Appl. 2024 Sep 17;17(9):e70005. doi: 10.1111/eva.70005 (PMC11408569; doi:10.1111/eva.70005)
Supplement: Supplementary file 2 — Appendix S2. [file EVA-17-e70005-s001.docx]

**Appendix 2 for:**

**Population genomics of the blue shark, *Prionace glauca*, reveals different populations in the Mediterranean Sea and the North East Atlantic**

*Agostino Leonea*, Sophie Arnaud-Haond, Massimiliano Babbucci, Luca Bargelloni, Ilaria Coscia, Dimitrios Damalas, Chrystelle Delord, Rafaella Franch, Fulvio Garibaldi, David Macias, Stefano Mariani, Jann Martinsohn, Persefoni Megalofonou, Primo Micarelli, Natacha Nikolic, Paulo A. Prodöhl, Emilio Sperone, Marco Stagioni, Antonella Zanzi, Alessia Cariani, Fausto Tinti**

#Filter initial Variant Calls

- Start with raw vcf file and change all genotypes with less than 5 reads to missing data
  This inital calls may be found on the dedicated GitHub repository
  The file needs to be unzipped before starting this workflow.

vcftools --vcf TotalRawSNPs.vcf --recode-INFO-all --minDP 5 --out BSdp5 --recode

**VCFtools - 0.1.14**

**(C) Adam Auton and Anthony Marcketta 2009**

**Parameters as interpreted:**

**--vcf TotalRawSNPs.vcf**

**--recode-INFO-all**

**--minDP 5**

**--out BSdp5**

**--recode**

**After filtering, kept 210 out of 210 Individuals**

**Outputting VCF file...**

**After filtering, kept 56004 out of a possible 56004 Sites**

**Run Time = 49.00 seconds**

- Now filter out all variants that are present below a minor allele frequency of 1% and are not called in at least 50% of samples

vcftools --vcf BSdp5.recode.vcf --recode-INFO-all --maf 0.01 -–max-missing 0.5 --out BSdp5g5 –recode

**(C) Adam Auton and Anthony Marcketta 2009**

**Parameters as interpreted:**

**--vcf BSdp5.recode.vcf**

**--recode-INFO-all**

**--maf 0.01**

**--max-missing 0.5**

**--out BSdp5g5**

**--recode**

**After filtering, kept 210 out of 210 Individuals**

**Outputting VCF file...**

**After filtering, kept 27863 out of a possible 56004 Sites**

**Run Time = 26.00 seconds**

- Now use a custom script called [filter_missing_ind.sh](https://github.com/jpuritz/dDocent/blob/master/scripts/filter_missing_ind.sh) to filter out bad individuals (there were a lot in this data set).

bash filter_missing_ind.sh BSdp5g5.recode.vcf BSdp5MI

At the prompt, enter yes and a custom cutoff of 0.60

yes

0.60

**VCFtools - 0.1.14**

**(C) Adam Auton and Anthony Marcketta 2009**

**Parameters as interpreted:**

**--vcf BSdp5g5.recode.vcf**

**--exclude lowDP.indv**

**--recode-INFO-all**

**--out BSdp5MI**

**--recode**

**Excluding individuals in 'exclude' list**

**After filtering, kept 210 out of 210 Individuals**

**Outputting VCF file...**

**After filtering, kept 27863 out of a possible 27863 Sites**

**Run Time = 23.00 seconds**

- Now use a second custom script [pop_missing_filter.sh](https://github.com/jpuritz/dDocent/blob/master/scripts/pop_missing_filter.sh) to filter loci that have high missing data values in a single population. You will need a file that maps individuals to populations popmap

./pop_missing_filter.sh BSdp5MI.recode.vcf popmap 0.25 0 BSdp5MIp25

**VCFtools - 0.1.14**

**(C) Adam Auton and Anthony Marcketta 2009**

**Parameters as interpreted:**

**--vcf BSdp5MI.recode.vcf**

**--keep keep.EATL**

**--out EATL**

**--missing-site**

**Keeping individuals in 'keep' list**

**After filtering, kept 35 out of 210 Individuals**

**Outputting Site Missingness**

**After filtering, kept 27863 out of a possible 27863 Sites**

**Run Time = 1.00 seconds**

**VCFtools - 0.1.14**

**(C) Adam Auton and Anthony Marcketta 2009**

**Parameters as interpreted:**

**--vcf BSdp5MI.recode.vcf**

**--keep keep.EMED**

**--out EMED**

**--missing-site**

**Keeping individuals in 'keep' list**

**After filtering, kept 57 out of 210 Individuals**

**Outputting Site Missingness**

**After filtering, kept 27863 out of a possible 27863 Sites**

**Run Time = 1.00 seconds**

**VCFtools - 0.1.14**

**(C) Adam Auton and Anthony Marcketta 2009**

**Parameters as interpreted:**

**--vcf BSdp5MI.recode.vcf**

**--keep keep.NATL**

**--out NATL**

**--missing-site**

**Keeping individuals in 'keep' list**

**After filtering, kept 30 out of 210 Individuals**

**Outputting Site Missingness**

**After filtering, kept 27863 out of a possible 27863 Sites**

**Run Time = 1.00 seconds**

**VCFtools - 0.1.14**

**(C) Adam Auton and Anthony Marcketta 2009**

**Parameters as interpreted:**

**--vcf BSdp5MI.recode.vcf**

**--keep keep.WMED**

**--out WMED**

**--missing-site**

**Keeping individuals in 'keep' list**

**After filtering, kept 88 out of 210 Individuals**

**Outputting Site Missingness**

**After filtering, kept 27863 out of a possible 27863 Sites**

**Run Time = 1.00 seconds**

**VCFtools - 0.1.14**

**(C) Adam Auton and Anthony Marcketta 2009**

**Parameters as interpreted:**

**--vcf BSdp5MI.recode.vcf**

**--exclude-positions loci.to.remove**

**--recode-INFO-all**

**--out BSdp5MIp25**

**--recode**

**After filtering, kept 210 out of 210 Individuals**

**Outputting VCF file...**

**After filtering, kept 23638 out of a possible 27863 Sites**

- Next, filter sites again my MAF, and filter out any sites with less than 90% overall call rate

vcftools --vcf BSdp5MIp25.recode.vcf --recode-INFO-all --maf 0.01 --max-missing 0.9 --out BSdp5MIp25g9 --recode

**VCFtools - 0.1.14**

**(C) Adam Auton and Anthony Marcketta 2009**

**Parameters as interpreted:**

**--vcf BSdp5MIp25.recode.vcf**

**--recode-INFO-all**

**--maf 0.01**

**--max-missing 0.9**

**--out BSdp5MIp25g9**

**--recode**

**After filtering, kept 210 out of 210 Individuals**

**Outputting VCF file...**

**After filtering, kept 22660 out of a possible 23638 Sites**

**Run Time = 19.00 seconds**

- Next, use a third custom filter script [dDocent_filters](https://github.com/jpuritz/dDocent/blob/master/scripts/dDocent_filters)

./dDocent_filters.sh BSdp5MIp25g9.recode.vcf BSdp5MIp25g9

Below is the included output:

This script will automatically filter a FreeBayes generated VCF file using criteria related to site depth, quality versus depth, strand representation, allelic balance at heterozygous individuals, and paired read representation.

The script assumes that loci and individuals with low call rates (or depth) have already been removed.

Contact Jon Puritz (jpuritz@gmail.com) for questions and see script comments for more details on particular filters

Number of sites filtered based on allele balance at heterozygous loci, locus quality, and mapping quality / Depth

2535 of 22660

Number of additional sites filtered based on overlapping forward and reverse reads

1855 of 20125

Is this from a mixture of SE and PE libraries? Enter yes or no.

no

Number of additional sites filtered based on properly paired status

211 of 18270

Number of sites filtered based on high depth and lower than 2*DEPTH quality score

976 of 18059

Histogram of mean depth per site


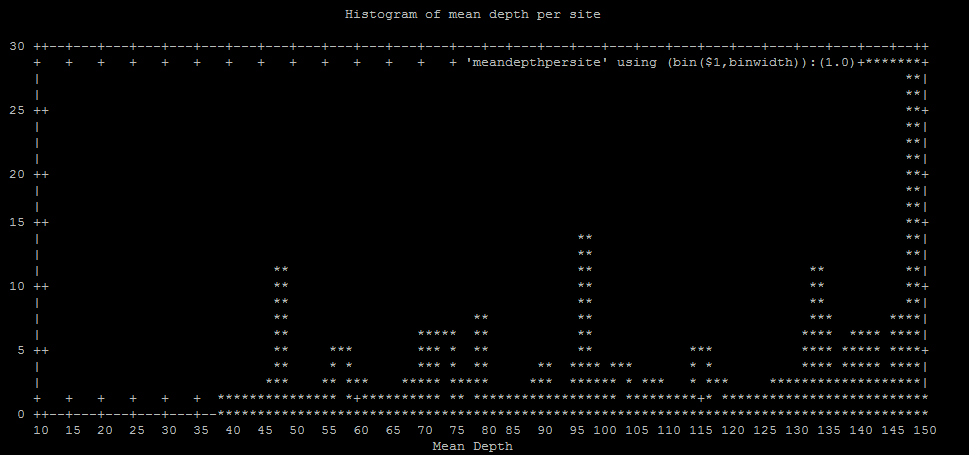


Same plot in excel


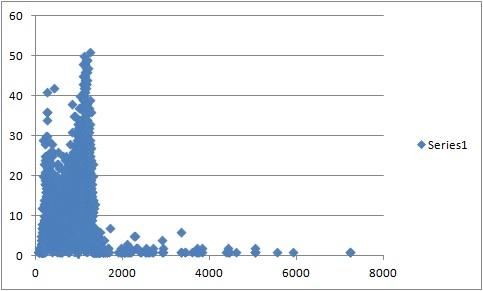


If distrubtion looks normal, a 1.645 sigma cutoff (~90% of the data) would be 310348.2065

The 95% cutoff would be 1255

Would you like to use a different maximum mean depth cutoff than 1255, yes or no

no

Number of sites filtered based on maximum mean depth

975 of 18059

Total number of sites filtered

5578 of 22660

Remaining sites

17082

Filtered VCF file is called Output_prefix.FIL.recode.vcf

Filter stats stored in BSdp5MIp25g9.filterstats

- Now, we need to break complex mutational events (combinations of SNPs and INDELs) into separate SNP and INDEL calls, and then remove INDELs.

vcfallelicprimitives -k -g BSdp5MIp25g9.FIL.recode.vcf | sed 's:\.|\.:\.\/\.:g' > BSdp5MIp25g9.prim

vcftools --vcf BSdp5MIp25g9.prim --recode-INFO-all --recode --out SNP.BSdp5MIp25g9 --remove-indels

**VCFtools - 0.1.14**

**(C) Adam Auton and Anthony Marcketta 2009**

**Parameters as interpreted:**

**--vcf BSdp5MIp25g9.prim**

**--recode-INFO-all**

**--out SNP.BSdp5MIp25g9**

**--recode**

**--remove-indels**

**After filtering, kept 210 out of 210 Individuals**

**Outputting VCF file...**

**After filtering, kept 16964 out of a possible 18102 Sites**

**Run Time = 15.00 seconds**

- Next, filter out loci that are out of HWE in more than half the populations, using [filter_hwe_by_pop.pl](https://github.com/jpuritz/dDocent/blob/master/scripts/filter_hwe_by_pop.pl) written by [Chris Hollenbeck](https://github.com/chollenbeck)

perl filter_hwe_by_pop.pl -v SNP.BSdp5MIp25g9.recode.vcf -p popmap -c 0.5 -o SNP.BSdp5MIp25g9HWE

**Processing population: EATL (35 inds)**

**Processing population: EMED (57 inds)**

**Processing population: NATL (30 inds)**

**Processing population: WMED (88 inds)**

**Outputting results of HWE test for filtered loci to 'filtered.hwe'**

**Kept 16872 of a possible 16964 loci (filtered 92 loci)**

- Restrict SNPs to loci only with 2 alleles.

vcftools --vcf SNP.BSdp5MIp25g9HWE.recode.vcf --recode-INFO-all --out SNP.BSdp5MIp25g9HWE2a --recode --max-alleles 2

**VCFtools - 0.1.14**

**(C) Adam Auton and Anthony Marcketta 2009**

**Parameters as interpreted:**

**--vcf SNP.BSdp5MIp25g9HWE.recode.vcf**

**--recode-INFO-all**

**--max-alleles 2**

**--out SNP.BSdp5MIp25g9HWE2a**

**--recode**

**After filtering, kept 210 out of 210 Individuals**

**Outputting VCF file...**

**After filtering, kept 16775 out of a possible 16872 Sites**

- Use [rad_haplotyper](https://github.com/jpuritz/WinterSchool.2016/blob/master/Exercises/Day%201/rad_haplotyper.pl) written by [Chris Hollenbeck](https://github.com/chollenbeck)

perl rad_haplotyper.pl -v SNP.BSdp5MIp25g9HWE2a.recode.vcf -p popmap -r reference.fasta -x 10 -mp 5

**Filtered 1635 loci below missing data cutoff**

**Filtered 192 possible paralogs**

**Filtered 0 loci with low coverage or genotyping errors**

**Filtered 0 loci with an excess of haplotypes**

This script uses called genotypes and aligned reads to make haplotype calls across RAD loci using both F and R reads.

- Move output and create a list of files that had high levels of missing data and potential paralogs

cp stats.out stats.out.HF

mawk '/Missi/' stats.out.HF | mawk '$9 > 30' > HF.missing

mawk '/para/' stats.out.HF > HF.para

cat HF.para HF.missing > HF.loci.tofilter

- Remove these loci entirely from the data set

./remove.bad.hap.loci.sh HF.loci.tofilter SNP.BSdp5MIp25g9HWE2a.recode.vcf

To see how many loci were retained:

mawk '!/#/' SNP.BSdp5MIp25g9HWE2a.filtered.vcf | wc -l

14729

Relatedness test with vcftools

vcftools --vcf SNP.BSdp5MIp25g9HWE2a.filtered.vcf --relatedness
