# Supplementary material for: Population Genomics of the Blue Shark, Prionace glauca, Reveals Different Populations in the Mediterranean Sea and the Northeast Atlantic
Source: Evol Appl. 2024 Sep 17;17(9):e70005. doi: 10.1111/eva.70005 (PMC11408569; doi:10.1111/eva.70005)
Supplement: Supplementary file 3 — Appendix S3. [file EVA-17-e70005-s002.pdf]

Rmarkdown for: Leone et al., Population genomics of the blue shark, Prionace glauca, reveals different populations in the Mediterranean Sea and the North East Atlantic. Evolutionary Applications

2024-06-04

If you want to knitr this markdown use: knittr::ops_chunk$set(echo = TRUE)

If using this Rmarkdown as a source of scripts/inspiration, please consider to cite the original paper:

Agostino Leone, Sophie Arnaud-Haond, Massimiliano Babbucci, Luca Bargelloni, Ilaria Coscia, Dimitrios Damalas, Chrystelle Delord, Rafaella Franch, Fulvio Garibaldi, David Macias, Stefano Mariani, Jann Martinsohn, Persefoni Megalofonou, Primo Micarelli, Natacha Nikolic, Paulo A. Prodöhl, Emilio Sperone, Marco Stagioni, Antonella Zanzi, Alessia Cariani, Fausto Tinti. Population genomics of the blue shark, *Prionace glauca*, reveals different populations in the Mediterranean Sea and the North East Atlantic, Evolutionary Applications

Sampling Design

First of all, let's clean the workspace:

```
rm(list = ls())
```

Then let's create a plot with the sampling design and specimens location:

```
library(rworldmap)
library(ggmap)
library(maps)
library(sp)
library(mapplots)
library(maptools)
library(raster)
library(RgoogleMaps)
library(ggOceanMaps)
library(RgoogleMaps)
library(dplyr)
library(marmap)
library(fossil)
```

```
library(geosphere)
library(ggspatial)

#load the csv file blu.csv with all specimen coordinates

dataset <-read.csv(file = "blu.csv", sep = ",")

NATL <- dataset %>% filter(
  area %in% c("NATL" )
)

EATL <- dataset %>% filter(
  area %in% c("EATL" )
)


WMED <- dataset %>% filter(
  area %in% c("WMED" )
)

EMED <- dataset %>% filter(
  area %in% c("EMED" )
)

#Then attach the datasets
attach(NATL)
attach(EATL)
attach(WMED)
attach(EMED)
#attach(EMED_O)

dt <- data.frame(lon = c(-20, -20, 30, 30), lat = c(30, 52, 30, 52))

basemap(data = dt, bathymetry = TRUE) + annotation_scale(location = "br") +
  annotation_north_arrow(location = "tr", which_north = "true") +
  geom_point(data = NATL, mapping = aes(x = lon, y = lat), color = "green") +
  geom_point(data = EATL, mapping = aes(x = lon, y = lat), color = "red") +
  geom_point(data = WMED, mapping = aes(x = lon, y = lat), color = "purple") +
  geom_point(data = EMED, mapping = aes(x = lon, y = lat), color = "blue")
```

To remove labels

```
basemap(data = dt, bathymetry = TRUE) + annotation_scale(location = "br") +
    annotation_north_arrow(location = "tr", which_north = "true") +
    geom_point(data = NATL, mapping = aes(x = lon, y = lat), color = "green") +
    geom_point(data = EATL, mapping = aes(x = lon, y = lat), color = "red") +
    geom_point(data = WMED, mapping = aes(x = lon, y = lat), color = "purple") +
    geom_point(data = EMED, mapping = aes(x = lon, y = lat), color = "blue") +
    labs(x = NULL, y = NULL)
#ggsave("Figure1_MAP.tiff", width = 12, height = 8, dpi = 300)
```

Population Genetics

Import genotype input file into genind object

first activate needed packages

```
library(adegenet)
library(OutFLANK)
library(qvalue)
library(dartR)
library(ggplot2)
library(ggforce)
library(hierfstat)
library(pegas)
library(poppr)
library(diveRsity)
library(SNPRelate)
library(graph4lg)
library(radiator)
library(assigner)
library(strataG)
library(stockR)
library(genepop)
library(dartR)
library(hierfstat)
library(pcadapt)
library(rnaturalearth)
library(ggspatial)
library(pegas)
library(poppr)
library(diveRsity)
library(grDevices)
library(MASS)
library(wesanderson)
library(plotly)
library(directlabels)
library(ggthemes)
library(devtools)
library(dartR)
library(brew)
library(RColorBrewer)
library(scales)
library(dplyr)
library(LEA)
library(gtools)
```

Import dataset and cleaning (Most of the filtering has been done in the server following a modified tutorial by Dr. Jon Puritz)

```
neutrals <- import2genind("neutralloci.gen")
```

```
#Update the site labels so that the site code is used.
# Use gsub to extract only letters from a vector

popNames(neutrals) = gsub("[^a-zA-Z]", "", popNames(neutrals))
popNames(neutrals)

coord<-read.table("coord.txt", header=TRUE, sep="\t")
neutrals@other$xy<-coord
#filter per NA
loci<-missingno(neutrals, type = "loci", cutoff = 0.10, quiet = FALSE,
          freq = FALSE)

neutrals<-missingno(loci, type = "geno", cutoff = 0.10, quiet = FALSE,
             freq = FALSE)
nLoc(neutrals)


#Calculate the percentage of complete genotypes per individuals.

locmiss_blues = propTyped(neutrals, by = "ind")
locmiss_blues[which(locmiss_blues < 0.80)] # print loci with < 80% complete genotypes

# Barplot
barplot(locmiss_blues, ylim = c(0,1),
        ylab = "Complete genotypes (proportion)",
        xlab = "Specimen", las = 2, cex.names = 0.7)
```

Check genotypes are unique

Check all individual genotypes are unique

```
#print multilocus genotype
mlg(neutrals)
#check if loci are polymorphic
isPoly(neutrals) %>% summary
```

Just in case:

duplicate_blues = mlg.id(neutrals) for (i in duplicate_blues){ # for each element in the list object if (length(duplicate_blues[i]) > 1){ # if the length is greater than 1 print(i) # print individuals that are duplicates } }

Just in case: Remove loci that are not polymorphic

poly_loci = names(which(isPoly(neutrals) == TRUE)) neutrals = neutrals[loc = poly_loci] isPoly(neutrals) %>% summary

Genetic Diversity and Differentiation

```
neuro <- gi2gl(neutrals, parallel = FALSE, verbose = NULL)
class(neuro)
FstWC84 <-  gl.fst.pop(neuro, nboots = 10000, percent = 95)
FstWC84$Fsts
FstWC84$Pvalues


result <- diveRsity::basicStats(
  infile = "neutralloci.gen",
  outfile = "BasicStat_neutro", fis_ci = TRUE, ar_ci = TRUE,
  fis_boots = 1000, ar_boots = 1000, mc_reps = 1000, rarefaction = FALSE,
  ar_alpha = 0.05, fis_alpha = 0.05)

gendiv.neutral <- data.frame(
  "NATL" = result$main_tab$NATL$overall,
  "EATL" = result$main_tab$EATL$overall,
  "WMED" = result$main_tab$WMED$overall,
  "EMED" = result$main_tab$EMED$overall
)

gendiv.neutral

write.table(gendiv.neutral, "gendiv.neutral.txt", sep="\t")
```

##PCA # Perform a PCA (principle components analysis) on the blueshark dataset.

Replace missing data with the mean allele frequencies and perform the PCA with dudi.pca

```
x = tab(neutrals, NA.method = "mean")
pca1 = dudi.pca(x, scannf = FALSE, scale = FALSE, nf = 4)
```

How much genetic variance is explained by each axis in percentage?

```
percent = pca1$eig/sum(pca1$eig)*100
barplot(percent, ylab = "% variance explained by eigenvectors", names.arg = round(percent, 1))
```

#Create the plot

```
#this PCA script was inspired to Jenkins et al. 2019
# Create a data.frame containing individual coordinates
ind_coords = as.data.frame(pca1$li)
# Rename columns of dataframe and add column with individuals
colnames(ind_coords) = c("PC1","PC2","PC3")
ind_coords$Ind = indNames(neutrals)

# Add a column with the site IDs
ind_coords$Site = neutrals$pop
```

```r
# Calculate centroid (average) position for each population
centroid = aggregate(cbind(PC1, PC2, PC3) ~ Site, data = ind_coords, FUN = mean)

# Add centroid coordinates to ind_coords dataframe
ind_coords = left_join(ind_coords, centroid, by = "Site", suffix = c("",".cen"))

# Define colour palette
cols = brewer.pal(nPop(neutrals), "Set1")
#in case of reordered genepop cols = c("#4DAF4A", "#E41A1C", "#984EA3", "#377EB8")
# Custom x and y labels
xlab = paste("PC1 (", format(round(percent[1], 1), nsmall=1)," %)", sep="")
ylab = paste("PC2 (", format(round(percent[2], 1), nsmall=1)," %)", sep="")

# Custom theme for ggplot2
ggtheme = theme(axis.text.y = element_text(colour="black", size=12),
                axis.text.x = element_text(colour="black", size=12),
                axis.title = element_text(colour="black", size=12),
                panel.border = element_rect(colour="black", fill=NA, size=1),
                panel.background = element_blank(),
                plot.title = element_text(hjust=0.5, size=15)
)

# Scatter plot axis 1 vs. 2
ggplot(data = ind_coords, aes(x = PC1, y = PC2))+
  geom_hline(yintercept = 0)+
  geom_vline(xintercept = 0)+
  # spider segments
  geom_segment(aes(xend = PC1.cen, yend = PC2.cen, colour = Site), show.legend = FALSE)+
  # points
  geom_point(aes(fill = Site), shape = 21, size = 3, show.legend = FALSE)+
  # centroids
  geom_label(data = centroid, aes(label = Site, fill = Site), size = 4, show.legend = FALSE)+
  # colouring
  scale_fill_manual(values = cols)+
  scale_colour_manual(values = cols)+
  # custom labels
  labs(x = xlab, y = ylab)+
  # custom theme
  ggtheme
# Export plot
# ggsave("Figure1.png", width = 12, height = 8, dpi = 600)
```

#Lets's try to remove the Eastern Mediterranean

```r
neutrals_noEMED <- neutrals[pop= c(1,3,4)]
# check if the EMED population has been removed
neutrals_noEMED$pop
```

#then redo the PCA with this subset

```r
x = tab(neutrals_noEMED, NA.method = "mean")
pca1 = dudi.pca(x, scannf = FALSE, scale = FALSE, nf = 3)
```

How much genetic variance is explained by each axis in percentage?

```
percent = pca1$eig/sum(pca1$eig)*100
barplot(percent, ylab = "% variance explained by eigenvectors", names.arg = round(percent, 1))
```

#Create the plot

```
# Create a data.frame containing individual coordinates
ind_coords = as.data.frame(pca1$li)
# Rename columns of dataframe and add column with individuals
colnames(ind_coords) = c("PC1","PC2","PC3")
ind_coords$Ind = indNames(neutrals_noEMED)

# Add a column with the site IDs
ind_coords$Site = neutrals_noEMED$pop

# Calculate centroid (average) position for each population
centroid = aggregate(cbind(PC1, PC2, PC3) ~ Site, data = ind_coords, FUN = mean)

# Add centroid coordinates to ind_coords dataframe
ind_coords = left_join(ind_coords, centroid, by = "Site", suffix = c("",".cen"))

# Define colour palette
cols = brewer.pal(nPop(neutrals_noEMED), "Set1")

# Custom x and y labels
xlab = paste("PC1 (", format(round(percent[1], 1), nsmall=1)," %)", sep="")
ylab = paste("PC2 (", format(round(percent[2], 1), nsmall=1)," %)", sep="")

# Custom theme for ggplot2
ggtheme = theme(axis.text.y = element_text(colour="black", size=12),
                axis.text.x = element_text(colour="black", size=12),
                axis.title = element_text(colour="black", size=12),
                panel.border = element_rect(colour="black", fill=NA, size=1),
                panel.background = element_blank(),
                plot.title = element_text(hjust=0.5, size=15)
)

# Scatter plot axis 1 vs. 2
ggplot(data = ind_coords, aes(x = PC1, y = PC2))+
  geom_hline(yintercept = 0)+
  geom_vline(xintercept = 0)+
  # spider segments
  geom_segment(aes(xend = PC1.cen, yend = PC2.cen, colour = Site), show.legend = FALSE)+
  # points
  geom_point(aes(fill = Site), shape = 21, size = 3, show.legend = FALSE)+
  # centroids
  geom_label(data = centroid, aes(label = Site, fill = Site), size = 4, show.legend = FALSE)+
  # colouring
  scale_fill_manual(values = cols)+
  scale_colour_manual(values = cols)+
  # custom labels
```

```
  labs(x = xlab, y = ylab)+
  # custom theme
  ggtheme
# Export plot
# ggsave("Figure1.png", width = 12, height = 8, dpi = 600)
```

#DAPC #Perform a DAPC (discriminant analysis of principal components) on the blueshark dataset. Before that, let's compute the optimal number of PCs to retain for the analysis

```
# Perform cross validation to find the optimal number of PCs to retain in DAPC
set.seed(123)
x = tab(neutrals, NA.method = "mean")
crossval = xvalDapc(x, neutrals$pop, result = "groupMean", xval.plot = TRUE)

# Check the number of PCs with the best statistics
crossval$`Root Mean Squared Error by Number of PCs of PCA`
crossval$`Number of PCs Achieving Highest Mean Success`
crossval$`Number of PCs Achieving Lowest MSE`
numPCs = as.numeric(crossval$`Number of PCs Achieving Lowest MSE`)
```

Run a DAPC using site IDs as priors

```
dapc1 = dapc(neutrals, neutrals$pop, n.pca = numPCs, n.da = 3)

# Analyse how much percent of genetic variance is explained by each axis
percent = dapc1$eig/sum(dapc1$eig)*100
barplot(percent, ylab = "Genetic variance explained by eigenvectors (%)", ylim = c(0,60),
        names.arg = round(percent, 1))
```

#Plot DAPC results

```
# Create a data.frame containing individual coordinates
ind_coords = as.data.frame(dapc1$ind.coord)

# Rename columns of dataframe
colnames(ind_coords) = c("Axis1","Axis2","Axis3")

# Add a column containing individuals
ind_coords$Ind = indNames(neutrals)

# Add a column with the site IDs
ind_coords$Site = neutrals$pop

# Calculate centroid (average) position for each population
centroid = aggregate(cbind(Axis1, Axis2, Axis3) ~ Site, data = ind_coords, FUN = mean)

# Add centroid coordinates to ind_coords dataframe
ind_coords = left_join(ind_coords, centroid, by = "Site", suffix = c("",".cen"))

# Define colour palette
```

```r
cols = brewer.pal(nPop(neutrals), "Set1")

# Custom x and y labels
xlab = paste("Axis 1 (", format(round(percent[1], 1), nsmall=1)," %)", sep="")
ylab = paste("Axis 2 (", format(round(percent[2], 1), nsmall=1)," %)", sep="")

# Scatter plot axis 1 vs. 2
ggplot(data = ind_coords, aes(x = Axis1, y = Axis2))+
  geom_hline(yintercept = 0)+
  geom_vline(xintercept = 0)+
  # spider segments
  geom_segment(aes(xend = Axis1.cen, yend = Axis2.cen, colour = Site), show.legend = FALSE)+
  # points
  geom_point(aes(fill = Site), shape = 21, size = 3, show.legend = FALSE)+
  # centroids
  geom_label(data = centroid, aes(label = Site, fill = Site), size = 4, show.legend = FALSE)+
  # colouring
  scale_fill_manual(values = cols)+
  scale_colour_manual(values = cols)+
  # custom labels
  labs(x = xlab, y = ylab)+
  ggtitle("DAPC")+
  # custom theme
  ggtheme
# Export plot
# ggsave("Figure3_DAPC.tiff", width = 12, height = 8, dpi = 300)
```

#We can also do a compoplot based on dapc1

```r
# tiff("compoplot.tiff", units="mm", width=180, height=90, res=600)
compoplot(dapc1, txt.leg=paste("Cluster", 1:4), lab="",
          xlab="individuals", col=cols)
# dev.off()
```

To see the contribution of SNPs on differentiation, we can have a look at the loading plot

```r
contrib <- loadingplot(dapc1$var.contr, axis = 2, thres = 0.0005, lab.jitter = 1)
contrib
```

#Looking at the Fst values, it's seems that the differentiation is related to the distance. Let's test for Isolation by Distance (IBD)

Let's try to use marmap package to extrapolate some spatial info of the study area

```r
papoue <- getNOAA.bathy(lon1 = -21, lon2 = 41,
lat1 = 25, lat2 = 55, resolution = 4, keep = FALSE)

# Creating color palettes
blues <- c("lightsteelblue4", "lightsteelblue3",
"lightsteelblue2", "lightsteelblue1")
greys <- c(grey(0.6), grey(0.93), grey(0.99))

plot(papoue, image = TRUE, land = TRUE, lwd = 0.03,
```

```
bpal = list(c(0, max(papoue), greys),
c(min(papoue), 0, blues)))
# Add coastline
plot(papoue, n = 1, lwd = 0.4, add = TRUE)

#you can explore different bathtymetry
bathyal <- get.area(papoue, level.inf = -4000, level.sup = -1000)
abyssal <- get.area(papoue, level.inf = min(papoue),
level.sup = -4000)
twilight <- get.area(papoue, level.inf = -1000, level.sup = -200)
ba <- round(bathyal$Square.Km, 0)
ab <- round(abyssal$Square.Km, 0)
tw <- round(twilight$Square.Km, 0)


plot(papoue, lwd = 0.2)
col.bath <- rgb(0.7, 0, 0, 0.3)
col.abys <- rgb(0.7, 0.7, 0.3, 0.3)
col.twil <- rgb(0.7, 0.8, 0.4, 0.3)
plotArea(bathyal, col = col.bath)
plotArea(abyssal, col = col.abys)
plotArea(twilight, col = col.twil)


legend(x="bottomleft",
legend=c(paste("bathyal:",ba,"km2"),
paste("abyssal:",ab,"km2"),
paste("twilight:",tw,"km2")),
col="black", pch=21,
pt.bg=c(col.bath,col.abys))

#you can load coordinates into the genind object
#for each specimen and then a transition object
#to be used by lc.dist to compute least cost distances
#between locations or just use specific location, as in this case.

#sites <- neutrals$other$xy

trans1 <- trans.mat(papoue)
trans2 <- trans.mat(papoue, min.depth = -50)


#IBD_per_pop_
WMED_coord <- WMED[c("lat","lon")]

WMED_coord$lat <- as.numeric(as.character(WMED_coord$lat))
WMED_coord$lon <- as.numeric(as.character(WMED_coord$lon))

center_point_WMED <- centroid(WMED_coord)
center_point_WMED

EMED_coord <- EMED[c("lat","lon")]
```

```
EMED_coord$lat <- as.numeric(as.character(EMED_coord$lat))
EMED_coord$lon <- as.numeric(as.character(EMED_coord$lon))

center_point_EMED <- centroid(EMED_coord)
center_point_EMED

EATL_coord <- EATL[c("lat","lon")]

EATL_coord$lat <- as.numeric(as.character(EATL_coord$lat))
EATL_coord$lon <- as.numeric(as.character(EATL_coord$lon))

center_point_EATL <- centroid(EATL_coord)
center_point_EATL

NATL_coord <- NATL[c("lat","lon")]

NATL_coord$lat <- as.numeric(as.character(NATL_coord$lat))
NATL_coord$lon <- as.numeric(as.character(NATL_coord$lon))

center_point_NATL <- centroid(NATL_coord)
center_point_NATL
################################################################################
WMED_points =  'lat,lon
     38.628388,15.599873
     44.249458,8.720920
     39.575049,2.079173
     36.520362,-2.986855'

WMED_points = read.table(text = WMED_points, sep = ',', header = TRUE, colClasses= rep('numeric', 2) )

center_point_WMED <- centroid(WMED_points)
center_point_WMED

EMED_points =  'lat,lon
     43.414454,14.438309
     39.750062,16.776286
     37.858648,15.621337
     35.104783,23.803859
     37.019347, 24.371546'

EMED_points = read.table(text = EMED_points, sep = ',', header = TRUE, colClasses= rep('numeric', 2) )

center_point_EMED <- centroid(EMED_points)
center_point_EMED


#all the AREAS estimated and the ADRIATIC

sites =  'x,y
     -12.14415,34.15676
     14.438309,43.414454
     -7.72219,51.44167
     7.29657,39.79052'
```

```
sites = read.table(text = sites, sep = ',', header = TRUE, colClasses= rep('numeric', 2) )

#all the AREAS estimated and the SOUTH OF CRETE

sites =  'x,y
     -12.14415,34.15676
     24.827703,34.539410
     -7.72219,51.44167
     7.29657,39.79052'

sites = read.table(text = sites, sep = ',', header = TRUE, colClasses= rep('numeric', 2) )


out1 <- lc.dist(trans1, sites, res = "path")
out2 <- lc.dist(trans2, sites, res = "path")


plot(papoue, xlim = c(-20, 40), ylim = c(25, 55),
     deep = c(-5000, -200, 0), shallow = c(-200, 0, 0),
     col = c("grey", "blue", "black"), step = c(1000, 200, 1), lty = c(1, 1, 1), lwd = c(0.6, 0.6, 1.2)
     draw = c(FALSE, FALSE, FALSE))
points(sites, pch = 21, col = "blue", bg = col2alpha("blue", .9),
       cex = 1.2)
text(sites[,1], sites[,2], lab = rownames(sites),
     pos = c(3, 4, 1, 2), col = "blue")
lapply(out1, lines, col = "orange", lwd = 5, lty = 1) -> dummy
lapply(out2, lines, col = "black", lwd = 1, lty = 1) -> dummy

#We can make a cleaned map

plot(papoue, xlim = c(-20, 40), ylim = c(25, 55), land = TRUE,
deep = c(-5000, -200, 0), shallow = c(-200, 0, 0),
col = c("grey", "blue", "black"), step = c(1000, 200, 1), lty = c(0, 0, 1), lwd = c(0.6, 0.6, 1.2),
draw = c(FALSE, FALSE, FALSE))
points(sites, pch = 21, col = "blue", bg = col2alpha("blue", .9),
cex = 1.2)
text(sites[,1], sites[,2], lab = rownames(sites),
pos = c(1, 1, 1, 1), col = "blue")
lapply(out1, lines, col = "orange", lwd = 5, lty = 1) -> dummy

#Then let's estimate the least cost distances
library(fossil)
dist0 <- round(earth.dist(sites), 0)
dist1 <- lc.dist(trans1, sites, res = "dist")
dist2 <- lc.dist(trans2, sites, res = "dist")

dist0
dist1
dist2
```

```
Dgeo <-read.table("DGEO.txt", header=TRUE, sep="\t")
Dgeo <- as.dist(Dgeo)
Dgeo
```

```
Dgen <-read.table("DGEN.txt", header=TRUE, sep="\t")
Dgen <- as.dist(Dgen)
Dgen



isolation_by_distance <- gl.ibd(
    distance = "Fst",
    coordinates = "xy",
    Dgen = Dgen,
    Dgeo = Dgeo,
    permutations = 20000,
    plot.out = TRUE,
    paircols = TRUE,
    save2tmp = FALSE,
    verbose = NULL
)
```

#Then we can try to estimate the relative migration rate

```
RelMigRate <- divMigrate(infile = "neutralloci.gen",
                         outfile = "relmigrate",
                         boots = 10000, stat = "all",
                         filter_threshold = 0,
                         plot_network = TRUE,
                         plot_col = "darkblue", para = 2)
```

#estimate migration rate using the private allele methods

```
Nm_private(
    inputFile = "neutralloci.gen",
    outputFile = "privAll",
    dataType = "Diploid",
    verbose = interactive()
)
```

#and then let's see these private alleles

```
poppr::private_alleles(neutrals, alleles ~ strata)
poppr::private_alleles(neutrals, alleles ~ strata, count.alleles = TRUE)
Pinfpriv <- poppr::private_alleles(neutrals, report = "data.frame")
ggplot(Pinfpriv) + geom_tile(aes(x = population, y = allele, fill = count))
```

This is an R Markdown document. Markdown is a simple formatting syntax for authoring HTML, PDF, and MS Word documents. For more details on using R Markdown see http://rmarkdown.rstudio.com.

When you click the **Knit** button a document will be generated that includes both content as well as the output of any embedded R code chunks within the document.

If using this Rmarkdown as a source of scripts/inspiration, please consider to cite the original paper:

Agostino Leone, Sophie Arnaud-Haond, Massimiliano Babbucci, Luca Bargelloni, Ilaria Coscia, Dimitrios Damalas, Chrystelle Delord, Rafaella Franch, Fulvio Garibaldi, David Macias, Stefano Mariani, Jann Martinsohn, Persefoni Megalofonou, Primo Micarelli, Natacha Nikolic, Paulo A. Prodöhl, Emilio Sperone, Marco Stagioni, Antonella Zanzi, Alessia Cariani, Fausto Tinti. Population genomics of the blue shark, *Prionace glauca*, reveals different populations in the Mediterranean Sea and the North East Atlantic, Evolutionary Applications
